# SHERLOCK4HAT: A CRISPR-based tool kit for diagnosis of Human African Trypanosomiasis

**DOI:** 10.1016/j.ebiom.2022.104308

**Published:** 2022-10-27

**Authors:** Núria Sima, Annick Dujeancourt-Henry, Blanca Liliana Perlaza, Marie-Noelle Ungeheuer, Brice Rotureau, Lucy Glover

**Affiliations:** a Trypanosome Molecular Biology, Department of Parasites and Insect Vectors, Institut Pasteur, Université Paris Cité, F-75015, Paris, France; b Trypanosome Transmission Group, Trypanosome Cell Biology Unit, INSERM U1201 & Department of Parasites and Insect Vectors, Institut Pasteur, Université Paris Sorbonne, Paris, France; c Institut Pasteur, ICAReB Platform (Clinical Investigation & Access to Research Bioresources) of the Center for Translational Science, Paris, France; d Parasitology Unit, Institut Pasteur of Guinea, Conakry, Guinea

**Keywords:** *Trypanosoma brucei*, HAT, Diagnostic, SHERLOCK

## Abstract

**Background:**

To achieve elimination of Human African Trypanosomiasis (HAT) caused by *Trypanosoma brucei gambiense* (gHAT), the development of highly sensitive diagnostics is needed. We have developed a CRISPR based diagnostic for HAT using SHERLOCK (Specific High-sensitivity Enzymatic Reporter unLOCKing) that is readily adaptable to a field-based setting.

**Methods:**

We adapted SHERLOCK for the detection of *T. brucei* species. We targeted *7SLRNA*, *TgSGP* and *SRA* genes and tested SHERLOCK against RNA from blood, buffy coat, dried blood spots (DBS), and clinical samples.

**Findings:**

The pan-Trypanozoon *7SLRNA* and *T. b. gambiense*-specific *TgSGP* SHERLOCK assays had a sensitivity of 0.1 parasite/μL and a limit of detection 100 molecules/μL. *T. b. rhodesiense*-specific *SRA* had a sensitivity of 0.1 parasite/μL and a limit of detection of 10 molecules/μL. *TgSGP* SHERLOCK and *SRA* SHERLOCK detected 100% of the field isolated strains. Using clinical specimens from the WHO HAT cryobank, the *7SLRNA* SHERLOCK detected trypanosomes in gHAT samples with 56.1%, 95% CI [46.25–65.53] sensitivity and 98.4%, 95% CI [91.41–99.92] specificity, and rHAT samples with 100%, 95% CI [83.18–100] sensitivity and 94.1%, 95% CI [80.91–98.95] specificity. The species-specific *TgSGP* and *SRA* SHERLOCK discriminated between the *gambiense*/*rhodesiense* HAT infections with 100% accuracy.

**Interpretation:**

The *7SLRNA*, *TgSGP* and *SRA* SHERLOCK discriminate between gHAT and rHAT infections, and could be used for epidemiological surveillance and diagnosis of HAT in the field after further technical development.

**Funding:**

Institut Pasteur (PTR-175 SHERLOCK4HAT), French Government's Investissement d’Avenir program Laboratoire d’Excellence Integrative Biology of Emerging Infectious Diseases (LabEx IBEID), and Agence Nationale pour la Recherche (ANR-PRC 2021 SherPa).

**Research in context**

**Evidence before this study**

We searched PubMed on March 29th 2022 with no language or date restrictions for studies that describe the development of new or improved diagnostics using the terms ‘*Trypanosoma brucei* CRISPR diagnostic’ or ‘*T. brucei gambiense* CRISPR diagnostic’ or ‘Neglected Tropical Disease CRISPR diagnostic’. We found one essay on the potential of CRISPR-Cas diagnostics for parasitic infections (Nanoscale, 2022, 14, 1885–1895), and one original research article on the development of CRISPR-dCas9 colorimetric diagnostic assay for *Leishmania* species (BioEssays, 44, e2100286).

**Added value of this study**

This study reports the development of a CRISPR-Cas13a molecular diagnostic for a trypanosome parasite species. This study details an original diagnostic toolbox that can detect both *T. brucei gambiense, T. brucei rhodesiense* and *T. brucei brucei*, and discriminate between these closely related species. This is a highly specific and sensitive new diagnostic assay that could meet the urgent needs highlighted by WHO for the diagnosis of gambiense Human African Trypanosomiasis.

**Implications of all the available evidence**

Human infection with *T. brucei gambiense* is included in the WHO roadmap to elimination, but this requires specific and high-sensitive diagnostic tools. This study describes the development of a new diagnostic that would aid in the elimination of infection with *T. brucei gambiense*.

## Introduction

Human African Trypanosomiasis (HAT), or sleeping sickness, is endemic to sub-Saharan countries and without prompt diagnosis and treatment, is usually fatal.^1^ It is caused by a tsetse-borne infection with protist parasites: *T. brucei (T. b.) gambiense* (gHAT), which represents 87% of the new cases and is endemic to central and western Africa, and *T. b. rhodesiense* (rHAT) which is responsible for the remaining 13% and is found in southern and eastern Africa. Due to both vector control, mass screening and treatment of those infected, the number of gHAT cases is decreasing, and has been maintained below 1000 new gHAT cases/year since 2018.^2^ In this context, gHAT has been included in the WHO roadmap to elimination, with zero transmission by 2030.^2^ Because of the preference of *T. b. rhodesiense* for the animal reservoir and the scarcity of control tools, the complete elimination of rHAT is not considered to be feasible.

The current diagnostic algorithms for gHAT rely on an initial serological test, followed by parasitological confirmation by direct observation under microscope, which is time consuming, requires trained staff and specialized equipment. The reduction in gHAT cases has brought about new challenges, not least that the positive predictive value of any diagnostic test diminishes as the disease burden is reduced. This has been already observed with the gHAT serological tests, the classical Card Agglutination Test for Trypanosomiasis (CATT) and the more recently developed rapid diagnostic tests (RDTs).^3^ Moreover, these tests are based on specific surface antigens, which if poorly or not expressed, can lead to missed diagnoses. No serological diagnostic tools are available for rHAT, and diagnosis is still based on clinical manifestations and visual detection of parasites by microscopy. Several molecular amplification tests have been developed for gHAT with promising results (18SrDNA-PCR, TBR-PCR, Tb177bp-qPCR, 18SrDNA-qPCR, SLRNA RT-qPCR, 18S RNA RT-qPCR, RIME-LAMP, 7SL-sRNA RT-qPCR)^4^, ^5^, ^6^ but their applications for mass screening are limited due to cost and required infrastructure. Importantly, diagnosis of gHAT is further complicated as there is increasing evidence that the traditional parasitological approaches fail to detect *T. b. gambiense* infections among ‘asymptomatic’ seropositive individuals who are apparently able to control infection to low levels and/or to maintain extravascular parasites, especially in the skin, in the absence of detectable blood parasitemia.^7^ Not only could these individuals contribute to transmission, but they may potentially go on to develop clinical gHAT.^7^, ^8^, ^9^ Ultimately, specific and highly-sensitive tools that are suitable for point-of-care (PoC) diagnosis and/or useable in a high-throughput mode in low-income countries are needed.

Adaptation of CRISPR technology towards the development of molecular diagnostics has led to highly sensitive and specific tools for the detection of *Plasmodium*,^10^^,^^11^ Zika, Dengue, SARS-CoV-2, Ebola and *Mycobacterium tuberculosis* to name a few.^12^ SHERLOCK (for Specific High-sensitivity Enzymatic Reporter unLOCKing) is a CRISPR-based approach that relies on the programable collateral nuclease activity of Cas enzymes to identify specific nucleic acid (NA) sequences in samples.^13^^,^^14^ Here, we describe the development of a point-of-care applicable, highly sensitive and specific diagnostic tool that can discriminate between trypanosome species causing g- and rHAT. We show its sensitivity and specificity using RNA from cultured parasites, simulated infections, field isolated samples and cryo-banked patient samples. Hence, after further technical improvement, our SHERLOCK4HAT tool kit would meet the current WHO recommendations for gHAT diagnostic.^15^^,^^16^

## Methods

### LwCas13a protein expression and purification

**Fig. S1:**
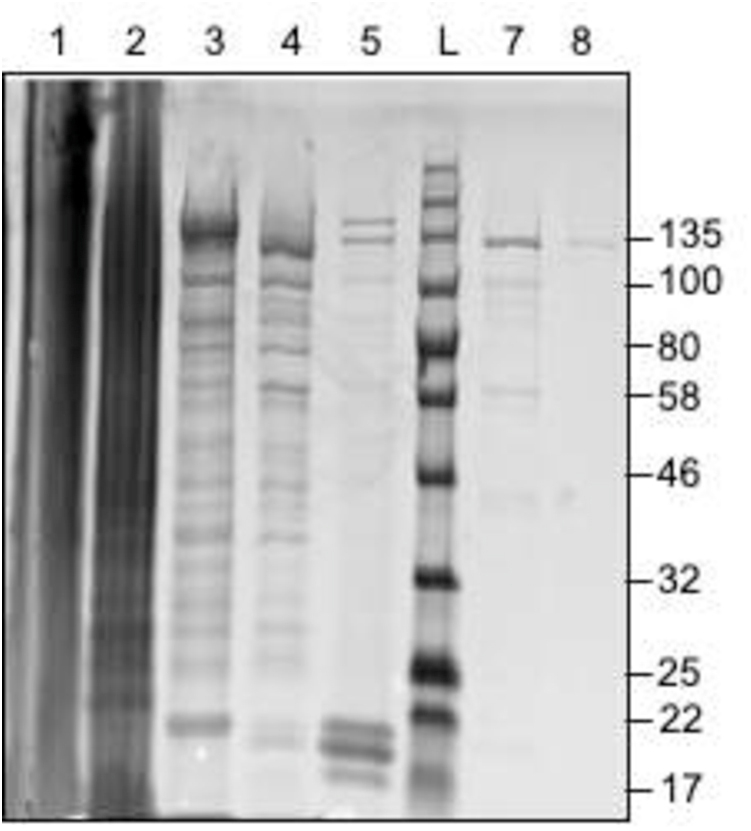
**LwaCas13a protein expression and purification.** Protein fractions collected during purification were analyzed by SDS-PAGE followed by Coomassie Blue staining. 1, cell pellet after clearing cell lysate; 2, cleared cell lysate supernatant; 3, flow-through following Strep-Tactin resin binding; 4, Strep-Tactin resin after SUMO protease cleavage; 5, eluted fraction post SUMO protease cleavage; L: Ladder; 7, eluted fraction after ion exchange chromatography (HiTrap column); 8, final product after size exclusion chromatography (Superdex column). The red ∗ at 135 kDa indicates the band corresponding to the purified LwaCas13a protein used in this study.

Plasmid pC013-Twinstrep-SUMO-huLwCas13a (RRID: Addgene_90097) was used to express LwCas13a in *Escherichia coli* Rosseta™ 2(DE3) pLySs competent cells. Cell pellet was lysed with supplemented lysis buffer (20 mM Tris–HCl pH8.0, 500 mM NaCl, 1 mM DTT, 100 mg lysozyme, 200U Deoxyribonuclease I) and LwCas13a protein was purified from the cleared supernatant as described in^17^ (Figure S1).

### Target selection and crRNA and RPA primer design

Target genes were selected as either unique or based on their conservation between *Trypanosoma spp.* using literature and publicly available data from TriTrypDB (https://tritrypdb.org/tritrypdb/app; RRID: SCR_007043). Candidate genes were aligned using BLAST with the Trypanosomatidae (taxid: 5654) nucleotide collection database from the National Center for Biotechnology Information (NCBI; RRID: SCR_006472). Alignments to ensure conservation of targets across the *Trypanozoon* subgenus or exclusivity between *T. brucei* subspecies was performed using Clustal Omega. Data available in TriTrypDB was used to identify SNPs found in different field isolates in the target genes *7SLRNA*, *SODB1* and *TgSGP*).^18^ To identify the SNPs in the *SRA* gene, the sequence variants AF097331, AJ345057, AJ345057^19^^,^^20^ were aligned using Clustal Omega and visualized in Jalview 2.11.1. RPA primers and crRNAs were designed to cover the conserved regions of the selected genes and outside of regions containing single nucleotide polymorphisms (SNPs). BLAST analysis with the nucleotide collection of all available genomes was performed to ensure RPA primers and crRNAs specific alignment. A 5′ T7 RNA polymerase promoter sequence (5′GAAATTAATACGACTCACTATAGGG) overhang was added to the RPA forward primers for *in vitro* transcription (IVT) during SHERLOCK reaction. The amplicons generated during RPA reactions are between 130 and 160 nt length. We used a 28 nt crRNA spacer for all guides in this study except for cr7SLbs which was 26 nt. The spacer sequence is joined to a 5′ direct repeat (DR) to generate the complete crRNA. To facilitate amplification from DNA templates a T7 RNA polymerase promoter sequence was added upstream of the crRNA (spacer + DR + T7 promoter 5′>3′). RPA primer, crRNA and DNA IVT template sequences are detailed in Table S1. SNPs identified for each target gene are included in Table S2.

### Target RNA and crRNA synthesis and purification

To produce the crRNA's, DNA IVT templates and T7-3G oligonucleotide were purchased from ThermoFisher. crRNAs were synthesized as described in^17^ with the following modifications. DNA IVT template (10 μM) and T7-3G oligonucleotide (10 μM) were annealed in standard Taq buffer (1×) by performing a 5-min denaturation, followed by slow cooling (ramp rate was adjusted to 0.13 °C/s) of the reaction to 4 °C in a PCR thermocycler (BioRad). IVT was performed using the HiScribe™ T7 Quick High Yield RNA Synthesis Kit (NEB E2050S), where 10 μL of annealed reaction were mixed with 10 μL of NTP buffer mix (NEB N2052A), 2 μL of T7 RNA polymerase mix (NEB M0255A) and 17 μL of RNase free water. The reaction was incubated for 6 h at 37 °C followed by 15 min of DNase I (M030A; 200 U/uL) digestion to remove DNA template. Purification of crRNA was done with Agencourt RNAClean XP beads (Beckman Coulter A63987) following the manufacturer's protocol and crRNA concentration was adjusted to 300 ng/μL. Urea Poly-Acrylamid Gel Electrophoresis were used to confirm the purity and correct size of crRNAs.

120 ng of RNA from *T. b. gambiense* ELIANE strain for *TgSGP* or 40 ng of RNA from *T. b. rhodesiense* EATRO strain for *SRA* RNA production were retro-transcribed using pT19 oligonucleotide and SuperScript IV Reverse Transcriptase (Thermo Fisher; 200 U/ul 18090200) following standard protocols. cDNAs were purified with Ampure XP (A63880) following manufacturer instructions and eluted in 30 μL of nuclease free water. *TgSGP* and *SRA* were amplified from 5 μL of cDNA, using TgSGP-FL-F and TgSGP-FL-R primers or SRA-FL-F and SRA-FL-R primers, respectively. For 7SLRNA production, the *7SLRNA* gene was amplified from 120 ng of *T. b. brucei* Lister 427 genomic DNA, using 7SLb-UP-F.6 and 7SLb-FL-R primers. The PCR amplification reaction was as follows: 0.2 mM dNTPs, 0.5 μM of each primer, 5 μL of DNA, 0.75 μL of DMSO, 0.5 μL of Phusion DNA polymerase (Thermo Scientific; F5302) in HF buffer (Thermo Scientific; F518) 1× in a 25 μL final volume and was run according to standard PCR settings. A T7 RNA polymerase promoter sequence overhang was included in each forward primer for *in vitro* transcription. IVT of the amplified genes was performed using the HiScribe™ T7 Quick High Yield RNA Synthesis Kit following manufacturer's instructions and the reaction incubated for 3 h at 37 °C followed by 15 min of DNaseI digestion. The single stranded RNA was purified using Agencourt RNAClean XP beads following the manufacturer's protocol. *In vitro* transcribed RNAs were sequenced, and Urea Poly-Acrylamid Gel Electrophoresis were used to confirm the purity and correct size of the target RNAs. Primer sequences used in this section are specified in Table S3.

### RNA isolation from cultured parasites

*T. b. brucei* Lister 427 bloodstream stage cells were cultured in HMI-11 medium (Gibco; Ref 074.9091JN) at 37.4 °C with 5% CO_2_. RNA was harvested at 1 × 10^6^ cell/mL. *T. b. gambiense* ELIANE and *T. b. rhodesiense* EATRO cell pellets were a gift from Annette MacLeod and *Leishmania major* cells were a gift from Gerald Spaeth. Total RNA from *T. b. brucei* Lister 427, *T. b. gambiense* ELIANE strain, *T. b. rhodesiense* EATRO strain, *L. major* and human embryonic kidney (HEK) 293T cells, was extracted with the RNeasy Mini kit (Qiagen).

### Simulated samples and total nucleic acid (TNA) extraction from blood, buffy coat and dried blood spots (DBS)

Blood from 10 healthy human donors was provided by ICAReB platform (Clinical Investigation & Access to Research Bioresources) in the Centre for Translational Science, at the Institut Pasteur (Paris).^21^ All participants gave written informed consent in the frame of the healthy volunteers Diagmicoll cohort (Clinical trials NCT 03912246) after approval of the CPP Ile-de-France I Ethics Committee (2009, April 30th). Whole blood was extracted in BD Vacutainer™ Glass ACD Solution Tubes and immediately processed. To determine the performance of *7SLRNA* SHERLOCK in whole blood, buffy coat and DBS, 2 × 10^5^ *T. b. brucei* Lister 427 parasites were spiked into 20 mL of human blood followed by 1:10 dilution to simulate parasitemia of 10 and 1 parasites/μL. Nine drops of 50 μL of each dilution of the simulated infected blood and non-infected blood were dried on Whatman 903™ Cards (GE Healthcare Life Science; Ref 10531018) and stored at RT for 24 h until processed. Three tubes of 500 μL of whole blood were snap-frozen and stored at −80 °C for 72 h. To obtain the buffy coat, 12 mL of each simulated infected blood dilution and non-infected blood were centrifuged at 1800 g for 10 min without brake, at 4 °C to prevent RNA degradation. Three tubes of 125 μL of buffy coat for each dilution were snap-frozen and stored at −80 °C for 72 h. TNA extraction from DBS was performed with the Nucleospin Triprep kit (Macherey–Nagel; Ref 740966.50). For that, 6 × 6 mm punches were incubated with 350 μL of RP1 buffer and 3.5 μL of β-mercaptoethanol at 37 °C for 30 min in agitation (1000 rpm). The instructions from the manufacturer were followed from this step onwards. DNA and RNA were eluted together in 40 μL of nuclease free water. TNAs from whole blood and buffy coat simulated samples was conducted with Maxwell RSC DNA blood kit (PROMEGA AS1400). The samples were pre-processed with 300 μL of lysis buffer and 30 μL of Proteinase K solution, vortexed for 10 s and incubated at 56 °C for 20 min. The volume was transferred to the Maxwell Cartridge and the kit protocol was run in the automated Maxwell RSC system. TNAs were eluted in 60 μL of elution buffer. Extractions for each dilution and type of sample were done in triplicate.

### Determination of analytical sensitivity

To determine the analytical sensitivity of *7SLRNA* SHERLOCK limiting dilutions of *T. b. brucei* Lister 427 parasites were spiked into un-infected human blood. TNAs were extracted from whole blood and buffy coat with Maxwell RSC DNA blood kit as detailed above. Three replicates of each dilution were assessed by SHERLOCK and the estimated LoD was determined as the lowest concentration where 3 out of 3 replicates were positive. The analytical sensitivity was confirmed in buffy coat by using 20 replicates of 0.66×, 1× and 1.5× the estimated LoD concentration. The analytical sensitivity was determined as the concentration where 95% of the samples gave positive results.

### Optimization of TNA extraction methods

To compare different TNA extraction methods from DBS, sheep blood was spiked with *T. b. brucei* Lister 427 parasites at limiting dilutions (1000-1 parasites/μL). Drops of 50 μL were dried into Whatman 903™ Cards and stored at RT for 24 h. For TNA extraction with RNeasy mini (Qiagen; Ref 74106) and micro kits (Qiagen; Ref 74004) 3 × 6 mm punches were resuspended with 370 μL of RLT buffer with 3.7 μL of β-mercaptoethanol and incubated at 37 °C for 30 min with agitation (1000 rpm). The punches and liquid were transferred into a QIAshredder column (Qiagen; Ref 79654) and spun at maxim speed for 1 min. The homogenized sample was then processed according to manufacturer's instructions without DNaseI digestion. DNA and RNA were eluted in the same fraction with 10 μL (RNeasy micro kit) or 30 μL (RNeasy mini kit) of nuclease-free water. For TNA extraction with the Nucleospin Triprep kit (Macherey–Nagel) 3 × 6 mm punches were resuspended with 350 μL of RP1 buffer with 3.5 μL of b-mercaptoethanol and incubated at 37 °C for 30 min in agitation (1000 rpm). The instructions from the manufacturer were followed from this step onwards and DNA and RNA were eluted together in 40 μL of nuclease free water. Maxwell RSC DNA blood kit (AS1400), Maxwell RSC SimplyRNA blood kit (AS1380) and RNeasy mini kit (Qiagen) were used to extract TNA from 250 μL or 125 μL of human buffy coat spiked with limiting dilutions of *T. b. brucei* Lister 427 parasites.

### RNA from the trypanosome collection at ITM

The 57 RNA samples used in this study were derived from trypanosome isolates representing different *Trypanosoma* species, subspecies, strains and growth conditions. For 9 strains, RNAs were extracted from different stabilates, either freshly isolated, or maintained in animal models and/or cultured in synthetic media with different durations and numbers of passages, and therefore theoretically presenting different transcriptomes. The collection contained 50 *T. b. gambiense* group 1 (46 bloodstream forms + 4 insect forms), 1 *T. b. gambiense* group 2, 2 *T. b. rhodesiense*, 1 *T. b. brucei*, 1 *Trypanosoma equiperdum* and 2 *Trypanosoma evansi* strains or clones. They were kindly provided by Nick Van Reet and Philippe Büscher (Institute of Tropical Medicine [ITM], Antwerp, Belgium) (Table S4).^22^ The RNA was kept at −80 and the concentration normalized to 5 ng/μL. Three microliters of input material were used for each SHERLOCK analysis. For assessing the specificity of SHERLOCK4HAT on the largest panel of different field species, strains and variants as possible, no initial sample size calculation was performed.

### Clinical samples

Clinical samples in this study were obtained from the WHO HAT Specimen biobank.^23^ They included buffy coats from 48 individuals living in *T. b. gambiense* endemic areas who were negative for gHAT by serology and parasitology, 50 patients with confirmed gHAT at stage 1, 48 patients with confirmed gHAT at stage 2, 20 individuals living in *T. b. rhodesiense* endemic areas who were negative for rHAT by serology and parasitology and 19 patients with confirmed rHAT at stage 2. Primary sample groups were defined by parasite species and parasitological results: 98 gHAT+, 62 gHAT−, 19 rHAT+ and 34 rHAT−. The sample size for gHAT (n = 160) is adapted to have 95% confidence and 80% power to detect a difference of 10% from expected sensitivity of 70% and specificity of 70%. The sample size for rHAT (n = 53) is adapted to have 95% confidence and 80% power to detect a difference of 10% from expected sensitivity of 95% and specificity of 95%.^24^^,^^25^ Blood from 14 healthy human donors was also provided as non-endemic negative controls by ICAReB platform (Clinical Investigation & Access to Research Bioresources) in the Center for Translational Science, at the Institut Pasteur (Paris).^21^ TNA were extracted from 125 μL of sample using the Maxwell RSC Blood DNA kit as detailed above. The material was eluted in 50 μL of elution buffer.

### SHERLOCK two-step reaction

For the isothermal amplification step, TwistAmp Basic kit (TwistDx; Ref TABA503KIT) was used according to manufacturer's instructions with the following modifications. For each reaction, 3 μL of input total nucleic acids (TNA) were incubated with 480 nM of each RPA primer (240 nM for *TgSGP* RPA primers), Reaction buffer 1×, 2.2 U of Transcriptor (Roche; Ref 03531287001), 1.5 U of Murine RNase inhibitor (NEB; Ref M0314L) and 14 mM MgOAc and 0.22 pellet of TwistAmp Basic kit, in a final volume of 11 μL. Reaction condition were optimized using different RPA primer concentrations (120, 240 or 480 nM) and MgOAc concentrations (14, 22 or 30 mM). The reactions were run using Hard-shell thin wall 96 well PCR Plates, sealed with Microseal ‘F’ Foil Seals (BioRad; Ref SP9601). Plates were incubated in a heating block set to 42 °C with thermoregulated lid. After a 5 min incubation, the plates were agitated for 15 s and the incubation resumed for 40 min. For the LwCas13a detection step, 1 μL of the previous reaction was incubated with 20 mM HEPES pH 6.5, 9 mM MgCl_2_, 1 mM rNTP mix (NEB), 126 ng of LwCas13a, 2 U of Murine RNase inhibitor (NEB), 25 U of NxGen T7 RNA Polymerase (Biosearch technology; Ref F8390L, 500 U/ul), 10 ng of crRNA and 125 nM of RNaseAlert probe V2 (Invitrogen) in a final volume of 20 μL. The reactions were run in 3 or 4 replicates in 384-well plates, F-bottom, μClear bottom, black, sterile, with lid (Greiner; Ref 781091). The incubations were maintained at 37 °C in the TECAN plate reader INFINITE 200 PRO Option M PLEX and the fluorescence was recorded at an initial time point and after 2 h 30 min or 3 h. For the lateral flow assay (LFA) readout Milenia HybriDetect strips (Ref 43009998) were used and the RNaseAlert probe (was substituted for 10 pmol of LF-RNA reporter (/56-FAM/rUrUrUrUrUrU/3Bio) and was incubated likewise. Following this, the SHERLOCK reaction was mixed with 80 μL of a PEG-based CRISPR-optimized Lateral Flow Assay Buffer (provided by Milenia Biotec GmbH, Germany). The strip was dipped in the mix and the results were interpreted after 5 min.

### SHERLOCK one-step reaction

For the single step SHERLOCK assay, 8 μL of input NA were mixed with 1 pellet of TwistAmp Basic kit, 20 mM HEPES pH 8, 60 mM KCl, 5% PEG-8000, 132 ng of LwCas13a in 1 mM Tris–HCl pH 7.5, 12 mM NaCl, 0.1% glycerol, 125 nM of RNaseAlert probe, 2 U/μL of ProtoScript II RT (NEB; Ref M03684), 0.1 U/μL of RNase H (NEB; Ref M02974), 1 U/μL of NxGen T7 RNA Polymerase (Biosearch technology; Ref F83904-1), 455 nM of each RPA primer, 10 nM of crRNA and 14 mM of MgOAc in a final volume of 107.5 μL. For each technical replicate, 20 μL of the mix were transferred to a 384-well plate, F-bottom, μClear bottom, black, sterile, with lid (Greiner). The incubations were done in the TECAN plate reader as described above. The fluorescence was monitored over 2 h 30 min at 37 °C with a 30 min interval between acquisitions.

### Quantitative PCR analysis

TNA were analyzed by qPCR using Luna Universal qPCR MasterMix (NEB; M3003L). The qPCR amplification mix contained 1 μL template and 0.4 μM of each primer (Tb177bp F/R). Reactions were run in triplicate in a Hard-shell PCR Plates 96 well, thin wall, which were sealed with Microseal ‘B’ Seals (BioRad; Ref MSF1001). All experiments were run on a CFX96 Touch Real-time Detection system with a C1000 Touch Thermal cycler (Bio-Rad), using the following PCR cycling conditions: 50 °C for 5 min, 95 °C for 10 min, then 40 cycles of 95 °C for 15 s and 66 °C for 1 min (fluorescence intensity data collected at the end of the last step), followed by a temperature gradient between 66 °C and 95 °C. The last step was used for dissociation analysis of the PCR product to monitor the amplicon identity. For that, the melt temperature of the amplicons from clinical samples was compared with that from *T. brucei* control nucleic acids. Sequence of primers in this section are listed in Table S3.

### Data analysis

We used the fluorescence given by the negative control, where water is used as input material, as the background fluorescence. To calculate the background-subtracted fluorescence intensities in a given multi-well plate, we subtracted the background fluorescence from each sample fluorescence at final time point. Using a ratio such as the fold-change over background fluorescence to normalize all results from all experiments allows inter-experimental comparisons without experiment-related biases. To calculate the fold-change over background fluorescence in each multi-well plate, sample fluorescence was divided by background fluorescence at final time point. For the optimization of the TNA extraction methods, fluorescence values were reported as fold-changes from the initial baseline fluorescence intensity by dividing the fluorescence value at last time point by the value at initial time point.

For the analysis of the clinical samples the following ratios were calculated for every target assessed:

• Negative template controls Ratio (Rntc) = Fold-change over the initial baseline fluorescence:

$$Rntc=\frac{FCntc\;t=3h}{FCntc\;t=0}$$

MeanRntc=meanRntcof3replicates

Where FC, fluorescence readout

• Positive template controls Ratio (Rpc) and sample Ratio (Rsample) = Fold-change over the background fluorescence at time 3h. Background fluorescence is given by the negative template control reaction:

$$Rpc=\frac{FCpc\;t=3h}{FCntc\;mean\;t=3h}$$

MeanRpc=meanRpcof3replicates

$$Rsample=\frac{FCsample\;t=3h}{FCntc\;mean\;t=3h}$$

MeanRsample=meanRsampleof3replicates

Where FC, fluorescence readout

**Fig. S2:**
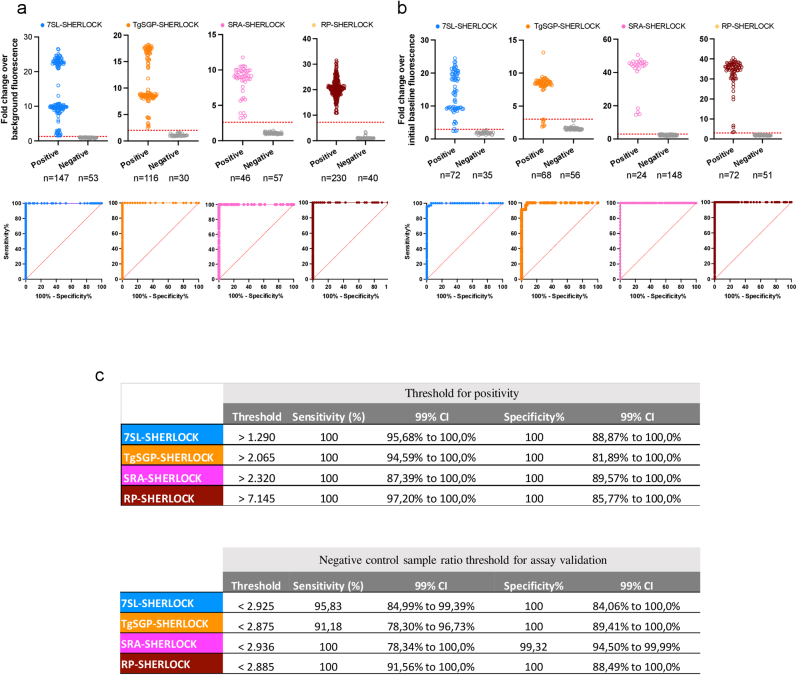
Receiver operating characteristic (ROC) curve analyses of positive and negative samples and threshold ratio calculation. (a), Laboratory generated positive and negative sample SHERLOCK ratios (fold change over background fluorescence) and ROC curves for *7SLRNA*, *TgSGP*, *SRA* and *RNase P* targets to calculate threshold for positivity. (b), Laboratory generated positive and negative sample SHERLOCK ratios (fold change over initial fluorescence) and ROC curves for *7SLRNA*, *TgSGP*, *SRA* and *RNase P* targets to calculate threshold ratio for assay validation. (c), Tables summarizing threshold ratios for each target and estimated sensitivity and specificity with the 99% CI.

Fluorescence ratios from positive and negative samples were computed by receiver operating characteristic (ROC) curve analysis for determining test positivity and validation thresholds (Figure S2).

**Fig. S3:**
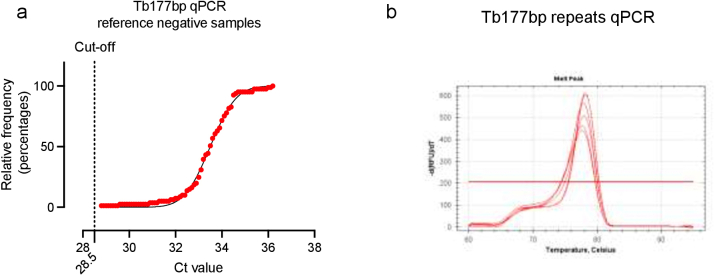
Criteria for validation qPCR reads. (a), Cumulative distribution of the Ct values from Tb177bp qPCR of 82 reference negative samples to stablish the Ct value cut-off. Samples were considered positive when the identity of the amplicon is been confirmed by dissociation analysis (specific melt temperature ±0.5°). The red dots represent the values of the empirical cumulative distribution function, while the black line represents its interpolation using the Gaussian smoothing proposed by GraphPad Prism 9.1.2. The Gaussian modelling of the cumulative distribution function was used to determine a threshold of Ct = 28.5 corresponding to an estimated probability of 0. The Gaussian approximation seems relevant as the overlap of the observations (in red) and the model (in black) is visually valid. (b), Dissociation analysis of the qPCR amplicons from a dilution series of TNA using cultured parasites. The specific melt temperature for the Tb177bp repeats amplicon is 78.

Technical replicates are performed on the same biological samples, whereas biological replicates are performed on distinct biological samples (different extractions). For qPCR, mean Ct values of 3 technical replicates were reported. A read was considered positive when the Ct value was lower than the established cut-off and the identity of the amplicon of these assays was confirmed by dissociation analysis (specific melt temperature ± 0.5°). The specific melt temperature for each amplicon was calculated by dissociation analysis of the qPCR amplicons using dilution series of TNA from cultured parasites. The Ct value cut-off was determined using cumulative distribution analysis (Figure S3).

### Statistical analysis

**Fig. 1:**
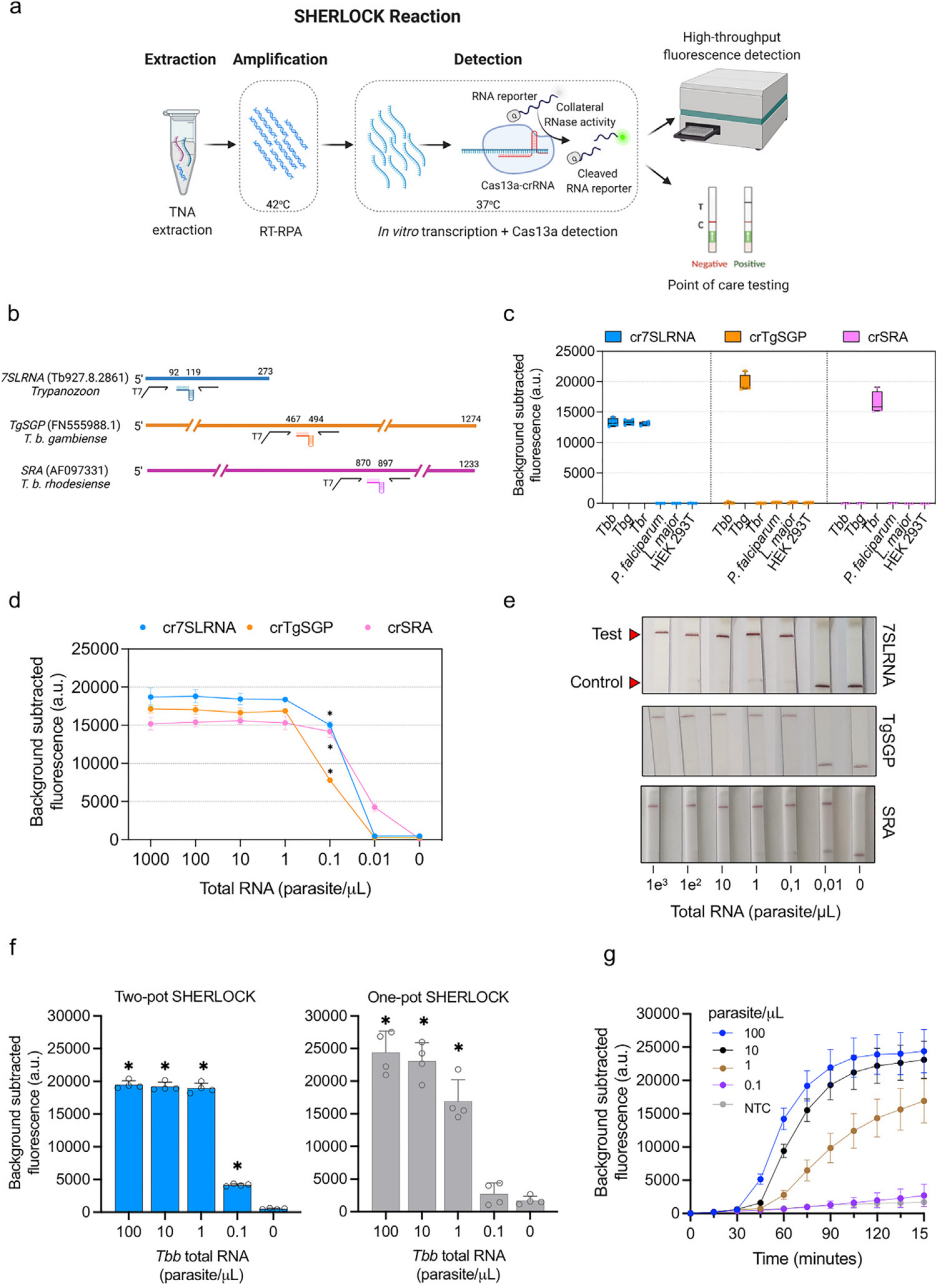
**Detection of *Trypanosoma brucei* sspp. RNA with SHERLOCK.** (a), Schematic overview of the SHERLOCK assay principle. Two-step SHERLOCK reaction is performed after TNA extraction. First, target NA is retro-transcribed and/or amplified during the RT-/RPA reaction at 42 °C. Second, the amplified target is *in vitro* transcribed and detected by Cas13a that cuts the RNA reporter upon target activation. Finally, the released reporter can be quantified with a fluorescence plate reader and/or with a LFA, making the methodology suitable for both mass screening and PoC testing. Panel created using BioRender.com. (b), Schematic showing selected target genes, RPA primer pairs and CRISPR guides. (c), Specificity of *7SLRNA*, *TgSGP* and *SRA* in a two-step SHERLOCK reaction using RNA from *T. b. brucei* Lister 427, *T. b. gambiense* ELIANE strain, *T. b. rhodesiense* EATRO strain, *Plasmodium falciparum*, *Leishmania major* and Human Embryonic Kidney (HEK) T cells. Fluorescence was measured after 150 min. Background subtracted fluorescence of 4 technical replicates is plotted as mean ± standard deviation (SD). a.u., arbitrary units. (d), Limits of detection of the *7SLRNA*, *TgSGP* and *SRA* targets in two-step SHERLOCK reactions. Dilution series of total RNA extracted from cultured parasites. *T. b. brucei* Lister 427, *T. b. gambiense* ELIANE strain, *T. b. rhodesiense* EATRO strain were used for the *7SLRNA*, *TgSGP* and *SRA* SHERLOCK reactions, respectively. Fluorescence was measured after 150 min. Coloured circles represent the mean ± SD of 4 technical replicates. Mann–Whitney test between fluorescence outputs of samples vs. no-template controls. ∗p < 0.05. a.u., arbitrary units. (e), Limit of detection of *7SLRNA*, *TgSGP* and *SRA* in a two-step SHERLOCK reaction with a lateral flow assay (LFA) read-out after 5 min. (f), Limits of detection of two-step vs. single-step *7SLRNA* SHERLOCK reactions on total RNAs from *T. b. brucei* Lister 427. Fluorescence was measured after 150 min. Blue bars represent the mean background subtracted fluorescence ± SD of 4 technical replicates shown as open circles. Mann–Whitney test between fluorescence outputs of samples vs. no-template controls. ∗p < 0.05. a.u., arbitrary units; a.u., arbitrary units. (g), Kinetics of the single step reaction in f. Each coloured circle represents the average of 4 technical replicates ± SD.

All plots and statistical analyses were performed with GraphPad Prism 9.1.2 (RRID: SCR_002798). Summary statistics on the values from 4 technical replicates are presented as mean ± standard deviation. Mann–Whitney tests between samples and controls were performed in Fig. 1, Fig. 3, Appendix A. To determine the Ct cut-off of the 177bp qPCR, we used the negative reference samples for HAT (n = 82; buffy coat samples from WHO). A Normal Linear Model of the cumulative distribution function was used to determine the threshold and the corresponding probability.

#### Ethics statement

Blood from healthy human donors was provided by ICAReB platform (Clinical Investigation & Access to Research Bioresources) in the Centre for Translational Science, at the Institut Pasteur (Paris). All participants gave written informed consent in the frame of the healthy volunteers Diagmicoll cohort (Clinical trials NCT03912246) after approval of the CPP Ile-de-France I Ethics Committee (2009, April 30th). For the WHO HAT Specimen biobank samples, approval was given by the WHO Ethical Review Committee, each national ethical committee where samples were taken and the national Ministries of Health.

### Role of the funding source

This study was funded by the Institut Pasteur (PTR-175 SHERLOCK4HAT), the French Government's Investissement d’Avenir program Laboratoire d’Excellence Integrative Biology of Emerging Infectious Diseases (LabEx IBEID), the Agence Nationale pour la Recherche Scientifique (ANR-PRC 2021 SherPa) and designed by study investigators. The funder had no role in this study.

## Results

### Selection of *Trypanosoma* target regions

**Fig. S4:**
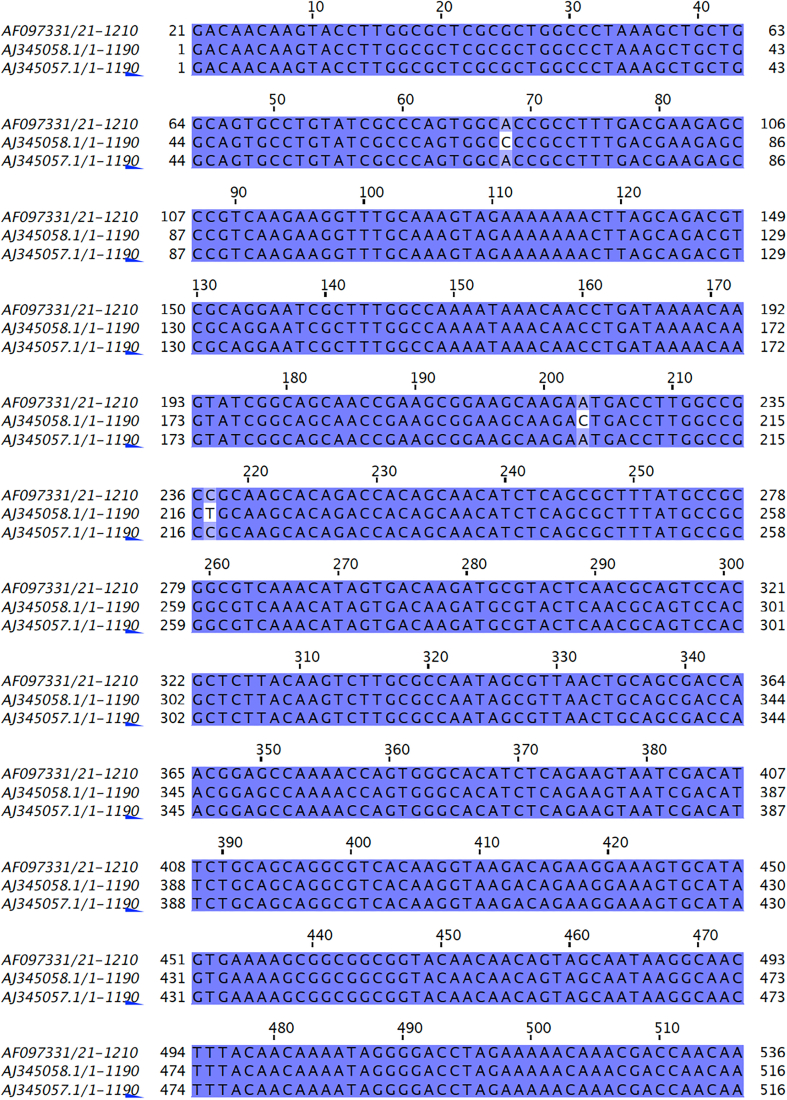
Comparison of *SRA* sequences AF097331, AJ345058.1 and AJ345057.1.

The SHERLOCK workflow combines isothermal recombinase polymerase amplification (RPA) with highly specific Cas13-CRISPR RNA target recognition coupled to readout via plate reader (for mass screening) or lateral flow strip (for PoC diagnosis) (Fig. 1a).^14^^,^^17^ To adapt SHERLOCK for the detection of *T. brucei* sp., we selected gene targets based on the following criteria (i) genes expressed in the human infective form of the parasites, (ii) *T. brucei* species- or subspecies-specific, (iii) degree of conservation between different strains and (iv) few to no single nucleotide polymorphisms (SNPs).^13^^,^^26^ We assessed four candidates out of seven genes matching these criteria, including the superoxide dismutase B1 (*SODB1*) gene (Tb927.11.15910), the component of the peptide recognition particle, *7SLRNA* (Tb927.8.2861), the *T. b. gambiense-*specific glycoprotein gene (*TgSGP*; FN555988.1) and the *T. b. rhodesiense-*specific serum resistance associated gene (*SRA*; AF097331). BLAST analysis revealed that the *SODB1* gene sequence is highly conserved between the *Trypanozoon* subgenus with 99% identity (*T. b. rhodesiense* sequence was not available) and shared certain degree of homology with *Trypanosoma cruzi* and *Leishmania donovani* genes (77.92% and 76.68% identity, respectively) (Data File S1). The *7SLRNA* is highly conserved within the *Trypanozoon* taxa, with 99% identity shared between the *T. brucei* sp., but more distant to the co-endemic species *Trypanosoma congolense* and *Trypanosoma vivax* with 86.31% and 79.77% identity respectively (Data File S1). *TgSGP* gene is specific to Group 1 *T. b. gambiense* and conserved across isolates in endemic territories.^27^^,^^28^ *TgSGP-*like genes, closely related to a possible ancestor *VSG* gene (Tb10.v4.0178), have been identified in other *T. brucei* sspp., with the 5ʹ region of the genes particularly conserved.^28^^,^^29^ *SRA* is specific to *T. b. rhodesiense* and provides an unbiased identification of the parasite.^19^^,^^30^, ^31^, ^32^, ^33^, ^34^, ^35^ Nevertheless, *SRA* is a *VSG*-like gene and the first 400 bp share 81% identity with a possible ancestor gene present in the *T. b. brucei* genome (Tb927.9.17380).^36^ Three *SRA* sequence variants (AF097331, AJ345057, AJ345057) have been identified from field isolates with homology from 97.9 to 99.7% (Figure S4).^19^^,^^20^^,^^34^ To develop a pan-*Trypanozoon* SHERLOCK diagnostic, RPA primers and CRISPR RNA guides (crRNAs) were designed to cover the conserved regions of the *7SLRNA* and *SODB1* genes in the *Trypanozoon* subgenus, that are distinct to the other trypanosomatids. For a *T. b. gambiense* and *T. b. rhodesiense* specific SHERLOCK diagnostic, guides were designed to the variable regions of *TgSGP* and *SRA* genes. In an attempt to ensure our tests would be applicable across a wide range of field isolated strains, identified SNPs were considered and guides were designed outside of these regions (Table S2).

### SHERLOCK distinguishes between the three *T. brucei* subspecies with high sensitivity

**Fig. S5:**
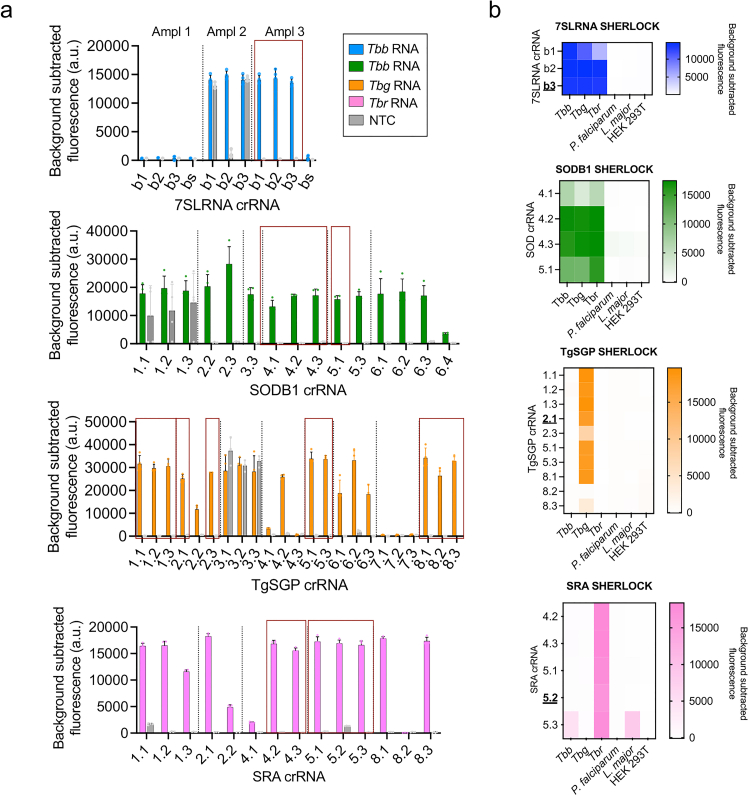
RPA primer pair and CRISPR RNA guide screening for *Trypanosoma bruc*ei spp. target genes. (a), SHERLOCK performance using multiple combinations of RPA primer pairs and crRNA covering *7SLRNA*, *SODB1*, *TgSGP* and *SRA* gene sequences. Bars represent the mean background subtracted fluorescence of 4 technical replicates +SD. Total RNA at 5 ng/mL from *T. b. b.* Lister 427, *T. b. g.* ELIANE strain or *T. b. r.* EATRO strain was used for the *7SLRNA* and *SODB1*, *TgSGP* and *SRA* reactions, respectively. Red boxes highlight the crRNA that were further analysed. (b), Specificity of selected RPA primers and crRNA guides was assessed using RNA from *T. b. b.* Lister 427, *T. b. g.* ELIANE strain, *T. b. r.* EATRO strain, *P. falciparum*, *L. major* and human embryonic kidney (HEK) 293T cells. In bolt, the selected guides for following experiments. NTC, non-template control; *Tbb*, *T. b. brucei*; *Tbg*, *T. b. gambiense*; *Tbr*, *T. b. rhodesiense*.

**Fig. S6:**
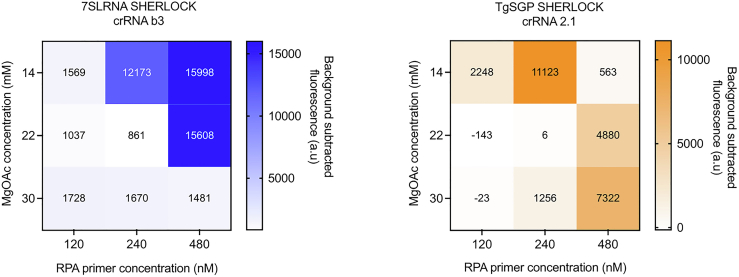
Heat maps of RPA primer and MgOAc concentration combinations to detect *7SLRNA* and *TgSGP* targets. RPA screens comprised of 9 set combinations of 3 MgOAc and 3 primer pair concentrations for best-performing condition to amplify sequence targets. Input ssRNA at 2000 aM for *7SLRNA* and 200 aM for *TgSGP*.

**Fig. S7:**
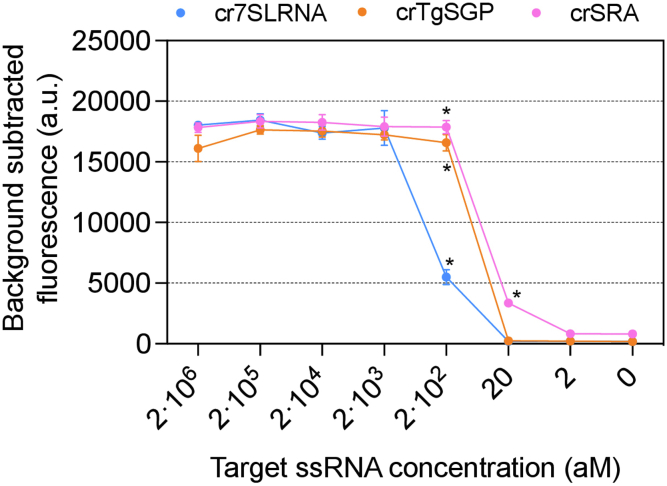
SHERLOCK limit of detection (LoD) in the attomolar range. Dilution series of i*n vitro* transcribed target *7SLRNA*, *TgSGP* and *SRA* RNAs were used for the *7SLRNA*, *TgSGP* and *SRA* SHERLOCK reactions respectively. Mann–Whitney test between fluorescence output of samples versus no-template controls. ∗p < 0.05.

We focused on developing a SHERLOCK diagnostic for the two subspecies of *T. brucei* that cause HAT. We screened several RPA primer pairs and crRNA combinations for each of the selected target genes (Figure S5 and Table S1 and S5). For *7SLRNA* target, 3 RPA amplicons (Ampl) were combined with 4 crRNA candidates (Ampl 1:crRNAb1-b3, bs, Ampl 2:crRNAb1-b3, Ampl 3:crRNAb1-b3, bs); for *SODB1* target, 6 RPA amplicons and 15 crRNA candidates were studied (Ampl 1:crRNA 1.1–1.3, Ampl 2:crRNA 2.2–2.3, Ampl 3:crRNA 3.3, Ampl 4:crRNA 4.1–4.3, Ampl 5:crRNA 5.1, 5.3, Ampl 6:crRNA 6.1–6.4); 8 RPA amplicons and 23 crRNA were tested for *TgSGP* (Ampl 1:crRNA 1.1–1.3; Ampl 2:crRNA 2.1–2.3; Ampl 3:crRNA 3.1–3.3, Ampl 4:crRNA 4.1–4.3, Ampl 5:crRNA 5.1, 5.3, Ampl 6:crRNA 6.1–6.3, Ampl 7:crRNA 7.1–7.3, Ampl 8:crRNA 8.1–8.3); and 5 RPA amplicons and 14 crRNA were assessed for *SRA* (Ampl 1:crRNA 1.1–1.3, Ampl 2:crRNA 2.1–2.2, Ampl 4:crRNA 4.1–4.3, Ampl 5:crRNA 5.1–5.3, Ampl 8:crRNA 8.1–8.3) (Figure S5 and Tables S1 and S5). A single RPA amplicon-crRNA combination was selected for each gene target based on highest signal-to-noise ratio (Figure S5a) and specificity (Figure S5b) when compared to target recognition in two co-endemic parasite species *L. major* and *Plasmodium falciparum* and to human embryonic kidney (HEK) T cells, as a representation of the host. Based on sensitivity and specificity results, we selected single combination of RPA primers and crRNA sequences for *7SLRNA*, *TgSGP* and *SRA* for all subsequent analyses (Fig. 1b and Table 1). The specificities were shown for *7SLRNA* as a pan-*Trypanozoon* diagnostic target, and *TgSGP* and *SRA* as species-specific diagnostic targets for *T. b. gambiense* and *T. b. rhodesiense*, respectively (Fig. 1c). The *7SLRNA* SHERLOCK was more sensitive than *SODB1* SHERLOCK, the second pan-*Trypanozoon* target, hence no further experiments were run with the *SODB1* target. We then wanted to determine the limit of detection (LoD) of each SHERLOCK reaction. The *SRA* SHERLOCK already showed high sensitivity (Fig. 1d) and did not require further optimisation. For the *7SLRNA* and *TgSGP* SHERLOCK reactions, we tested various RPA primer and Magnesium oxalacetate (MgOAc) concentrations (Figure S6), as both can have a direct impact on the amplification rate, and thus on the amplification efficiency. For all subsequent analysis, 480 nM of RPA primer and 14 mM MgOAc were used in the *7SLRNA* SHERLOCK reaction and 240 nM RPA primer and 14 mM MgOAc in the *TgSGP* SHERLOCK reaction (Figure S6). Using input RNA extracted from cultured parasites, the LoD for the *7SLRNA*, *TgSGP* and SRA targets was determined to be 0.1 parasite/μL (Fig. 1d), which falls within the range of parasitemia commonly observed in HAT patients.^37^^,^^38^ Using *in vitro* transcribed RNA, the LoD was calculated to be 200 aM (100 molecules/μL) for *7SLRNA* and *TgSGP*, and 20 aM (10 molecules/μL) for *SRA* (Figure S7).

**Table 1:** RPA primers and crRNAs selected in this study.

Trypanosoma species target (accession number)	RPA forward primer with *T7 promoter* overhang 5ʹ>3ʹ	RPA reverse primer 5ʹ>3ʹ	crRNA sequence 5ʹ>3ʹ (DR + spacer 28 nt)
*Trypanozoon* 7SLRNA (Tb927.8.2861)	*GAAATTAATACGACTCACTATAGG* *G*CGGAGCGCATTGCTCTGTAACCTTC	CCACTTTAACGGCGCGAGAACGCC	GAUUUAGACUACCCCAAAAACGAAGGGGACUAAAACCAACACCGACACGCAACCAAGCAGAACA
*T. brucei gambiense* TgSGP (FN555988.1)	*GAAATTAATACGACTCACTATAGGG*TTT GACAGCATGGGAGATGCAACTCGCAAG	CAAGTCCGTGACAGCCTTGCCCGTTCCCGC	GAUUUAGACUACCCCAAAAACGAAGGGGACUAAAACAAAUGGCUUCCAGCUUCCGCUGUGCUAG
*T. brucei rhodesiense* SRA (AF097331)	*GAAATTAATACGACTCACTATAGGG*AA AAGCAAACTCGTATCAATCCAAGAGT	CCCTTGTCTTTGTCGTCGCCAATTGCGT	GAUUUAGACUACCCCAAAAACGAAGGGGACUAAAACGGCGACCUGCUGCUUGCAGCUUUCAGUG

### SHERLOCK can be adapted to a PoC diagnostic use

SHERLOCK is amenable to readout by lateral flow assay (LFA).^14^ Importantly, using a polyethylene glycol (PEG)-based CRISPR-optimized buffer (provided by Milenia Biotec), we were able to detect the *7SLRNA*, *TgSGP* and *SRA* SHERLOCK targets with the same respective sensitivities as with the fluorescent readout, but with a reduced background signal as compared to the commercially available LFA buffers, thereby reducing the ambiguity of the readout (Fig. 1, Appendix A).

**Fig. S9:**
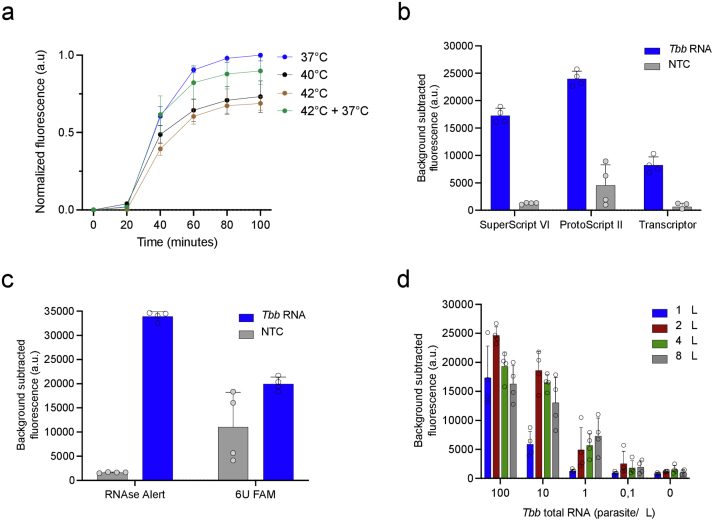
Optimization of one-tube reaction. (a), Comparison of different incubation temperatures. Three different temperatures were assessed, 37 °C, 40 °C and 42 °C, and a combination of 42 °C for 10 min (optimal for RT-PCR) followed by 37 °C for the rest of the reaction (optimal for Cas13a). Incubation at 37 °C resulted to be the best performance temperature, in terms of speed and signal. RNA from 100 parasite/mL was used in each reaction. (b), Retro transcriptase enzymes from different manufacturers were compared. ProtoScript II from NEB was selected for the optimized reaction as it gave higher signal and was more cost-effective. RNA from 100 parasite/mL was used in each reaction. (c), Two RNA quenched fluorescent RNA reporters were tested. RNase Alert was selected for the optimized reaction as it led to a higher signal-to-noise ratio. RNA from 100 parasite/mL was used in each reaction. (d), Different input volumes were assessed in the one-tube reaction. For the optimized reaction 8 mL of input volume was used to increase the sensitivity of the test.

To optimize SHERLOCK4HAT as a one-tube reaction,^10^^,^^39^ we focussed on the *7SLRNA* target and modified the reaction components and conditions (Figure S9). Given that the reverse-transcriptase (RT) and Cas13 activities have different temperature preferences, we evaluated the performance of one-tube reactions at temperatures from 37 °C to 42 °C, and we found that reactions at 37 °C had higher signal with reduced sample-to-result time (Figure S9a).

An additional consideration for the development of a PoC diagnosis for use in low-income countries is affordability. We therefore tested three RT enzymes from different manufacturers and selected ProtoScript II (NEB) as the most cost–effective reaction with a cost of 2.2 €/reaction (Figure S9b and Table S6). Given that Cas13a has uridine-cleavage preference,^14^^,^^40^ we compared RNase Alert with a 6U-FAM reporter. The signal intensity obtained with the 6U-FAM reporter was lower and was more prone to spontaneous degradation, as seen with the non-template control reaction (Figure S9c). Thus, we selected RNase Alert as a reporter for an optimized one-tube SHERLOCK diagnostic and used 8 μL of input material (Figure S9d). With these improvements, the *7SLRNA* one-tube SHERLOCK reaction had similar sensitivity than the two-step reaction and detected 1 parasite/μL in 1 h (Fig. 1f and g).

### The SHERLOCK4HAT diagnostic kit can accurately detect a trypanosome isolate across multiple regions and over extended periods of time

**Fig. 2:**
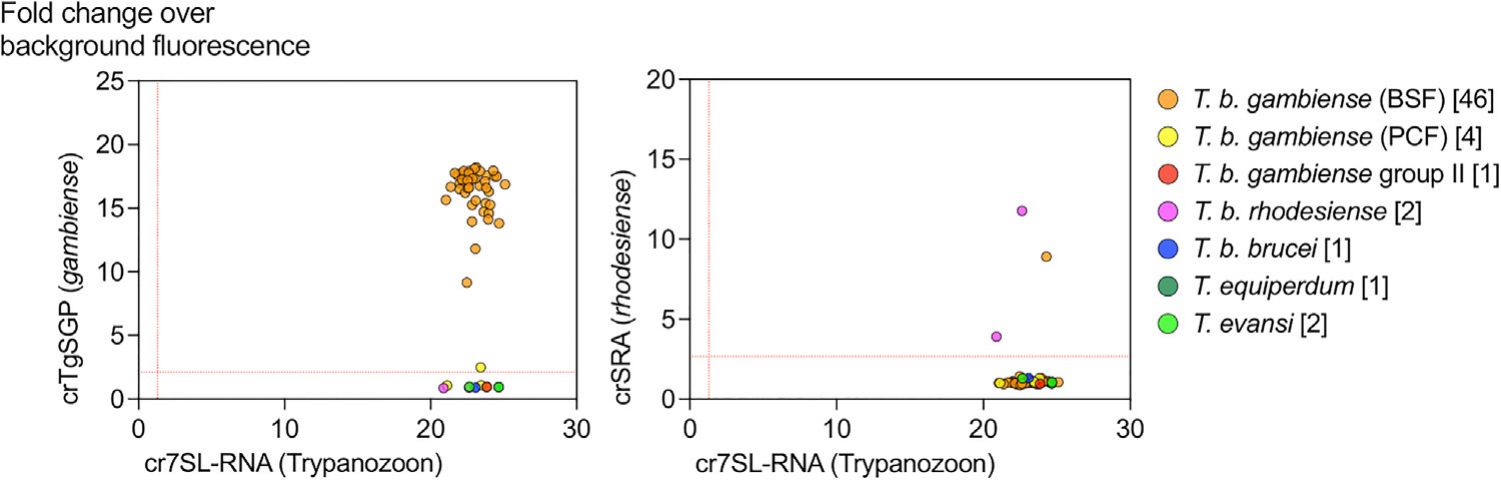
**Validation of SHERLOCK4HAT using field isolated samples.** SHERLOCK detection of *7SLRNA*, *TgSGP* or *SRA* targets using RNA extracted from field isolated trypanosome strains. *TgSGP* (left panel) and *SRA* (right panel) target readouts are plotted against *7SLRNA* readouts in fold change over background fluorescence. Each dot represents the average readout of 4 technical replicates. The thresholds for each target (red lines) were determined using ROC curve analyses of positive and negative sample data. In brackets, after the name of the species, number of strains analysed.

**Fig. S10:**
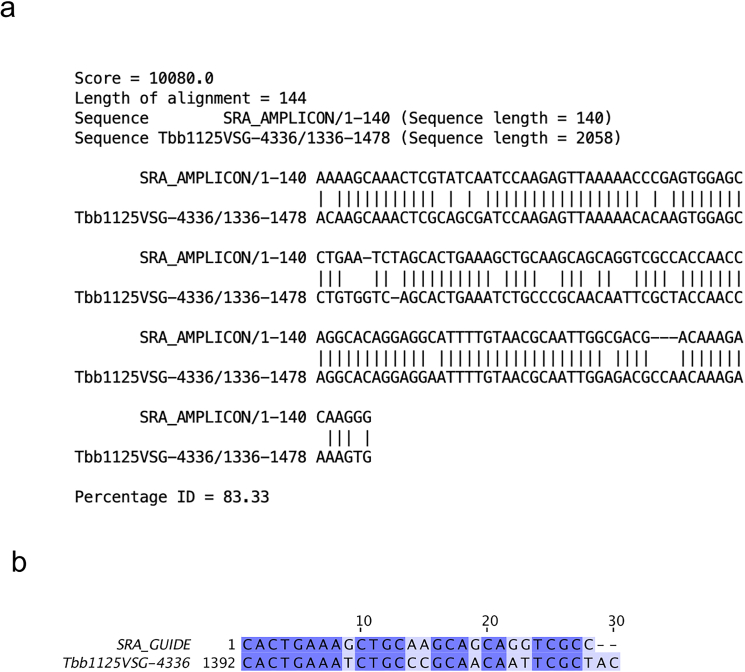
Alignment of *SRA* amplicon and guide with Tbb1125VSG-4336. (a), Clustal alignment of *SRA* SHERLOCK amplicon with Tbb1125VSG-4336 expressed in the triple positive field isolated sample AnTat 22.1. (b), Nucleotide mismatches between the *SRA* crRNA and Tbb1125VSF-4336.

Genetic variability between field isolates can potentially lead to false negatives with molecular diagnostic. Therefore, the development of a robust diagnostic hinges upon the ability to detect all parasite strains or variants. To demonstrate the robust specificity of our SHERLOCK4HAT diagnostic kit, we analyzed total RNAs from 57 *Trypanozoon* strains, isolated from their host over the course of 50 years and maintained at the Institute of Tropical Medicine (ITM, Antwerp, Belgium) (Table S4).^22^ Using our two-step SHERLOCK assay, all samples were positive for *7SLRNA*, confirming *7SLRNA* SHERLOCK as a pan-*Trypanozoon* diagnostic and epidemiological tool (Fig. 2 and Table S4). Within this set, the 46 *T. b. gambiense* Group 1 mammalian stage isolates tested positive for *TgSGP*, and 3 out of 4 *T. b. gambiense* Group 1 insect stage isolates were negative, as expected since *TgSGP* is only expressed in the mammalian stage of the parasite.^27^ The single *T. b. gambiense* Group 1 insect stage isolate (MHOM/CI/91/SIQUE1623) that was positive for *TgSGP*, may have retained low level expression of the gene. The 7 non-Group 1 *T. b. gambiense* strains tested negative for *TgSGP*, including the *T. b. gambiense* Group 2 sample (Fig. 2, and Table S4), thus confirming the diagnostic specificity of the *TgSGP* SHERLOCK for *T. b. gambiense* Group 1. The two *T. b. rhodesiense* strains included in the collection were positive for *SRA,* and 54 of the 55 non-*T. b. rhodesiense* strains tested negative for *SRA* (Fig. 2 and Table S4). A single isolate, AnTat 22.1, classified as *T. b. gambiense* Group 1 was positive for both *TgSGP* SHERLOCK and *SRA* SHERLOCK (Table S4). Sequence analysis showed that the *SRA* SHERLOCK target amplicon shared 83.3% identity with a *VSG* (Tbb1125VSG-4336, accession number: KX700900) that was expressed in this sample (Figure S10a). There are 7 nucleotide mismatches between the *SRA* guide and the homologous region in the *VSG* sequence (Figure S10b), and this is the most likely source of the cross-reactivity. In spite of the 1.8% cross-reactivity observed with the *SRA* SHERLOCK within the group of samples analyzed, 100% of the strains were detected with the corresponding test, confirming the specificity of *TgSGP* SHERLOCK and *SRA* SHERLOCK for diagnosis across endemic regions.

### SHERLOCK4HAT detects trypanosomes in dried blood spots, whole blood and buffy coat

**Fig. 3:**
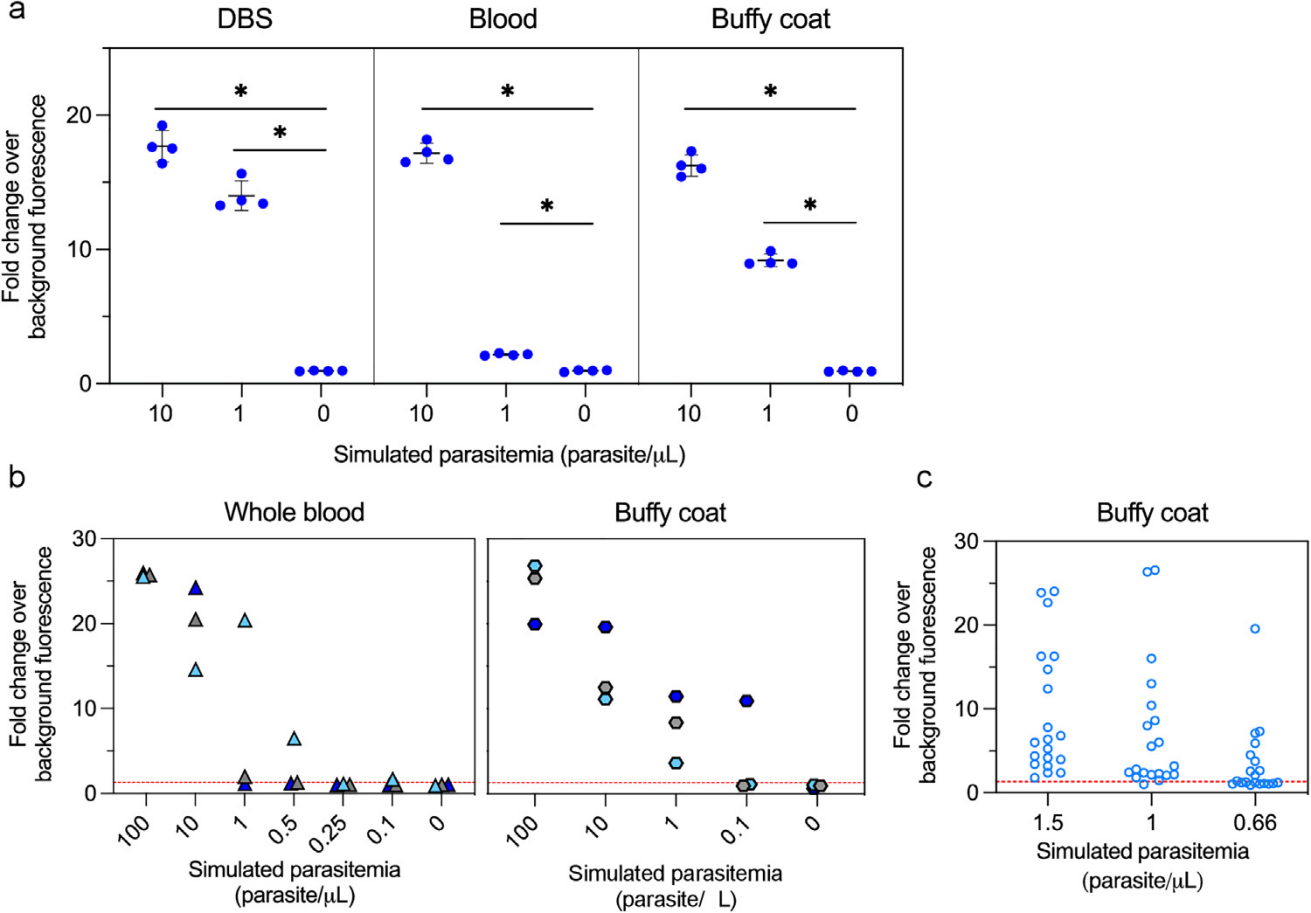
**Performance of SHERLOCK4HAT on dried blood spots, whole blood and buffy coat.** (a), A comparison of the performance of the *7SLRNA* SHERLOCK on trypanosome RNA extracted from dried blood spots (DBS), whole blood and buffy coat. All experiments were done in 4 replicates from a single pool of simulated infected blood. Fold changes over background fluorescence were plotted as mean ± SD. Mann–Whitney test between readout of samples vs. no-template controls. ∗p < 0.05. (b), Samples with known parasitemia were used to assess the analytical sensitivity of the *7SLRNA* SHERLOCK. Three replicates of each dilution were tested. The tentative limit of detection (LoD) was the lowest concentration where 3/3 replicates were positive for the test. The detection threshold (red line) was determined using ROC curve analyses of positive and negative sample data. (c), The LoD was confirmed by using samples at 0.66×, 1× and 1.5× the estimated LoD concentration of the buffy coat only. The experiment was done on 20 replicates and the LoD was determined to be the concentration at which 95% of the samples were positive for the test. The detection threshold (red line) was determined using ROC curve analyses of positive and negative sample data.

**Fig. S11:**
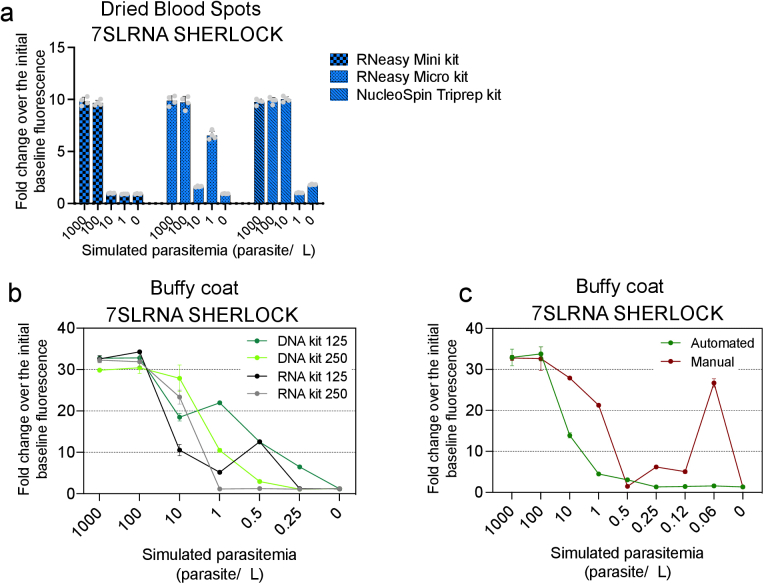
Total nucleic acid extraction optimization. (a), Comparison of TNA extraction from DBS. Fifty microliters of spiked sheep blood was dried on Whatman 903™ Cards and stored for 24 h at room temperature. TNA were extracted from 3 punches of 6 mm using the RNeasy mini kit (QIAGEN), RNeasy micro kit (QIAGEN) or the NucleoSpin Triprep (Macherey–Nagel). Bars represent mean readout + SD of 4 SHERLOCK replicates, shown as grey circles. (b), Trypanosome TNAs extraction from spiked human buffy coat using Maxwell automated system. Two Maxwell RSC kits (simplyRNA blood kit and Blood DNA kit) and two different input volumes (125 μL and 250 μL) were compared. (c), SHERLOCK assay for *7SLRNA* target. Uninfected human blood was spiked with 1000 p/μL followed by dilution series. Buffy coat was obtained by centrifugation and TNAs were purified either with Maxwell automated system (Maxwell RSC Blood DNA kit) or with the manual column-based QIAGEN RNeasy mini kit, with minor modifications (see materials and methods). The data from the Maxwell extraction system is also shown in Fig. 3b.

To optimize the *7SLRNA* SHERLOCK for epidemiological surveys, we compared three methods of TNA extraction from DBS using non-infected sheep blood spiked with cultured *T. brucei* parasites spotted on Whatman 903™ Cards. Our *7SLRNA* SHERLOCK was able to detect 100 parasites/μL using a RNeasy kit (Qiagen) and 10 parasites/μL with the NucleoSpin Triprep kit (Macherey–Nagel) (Figure S11a). We saw consistently greater sensitivity with the NucleoSpin Triprep kit, and therefore used it for subsequent extractions from DBSs. Mass screening campaigns are expected to result in a high volume of samples that require subsequent processing, thus, an automated system that minimizes the hands-on time in the extraction process and the cross-contamination between samples is preferred. We compared the TNA extraction performance on buffy coat of two different kits from the automated, paramagnetic beads-based system Maxwell RSC (Promega) and the manual column-based system from Qiagen (Figure S11b and c). Maxwell RSC DNA blood kit was more efficient than Maxwell RSC RNA kit for TNA extraction using simulated infected samples and showed no cross-contamination, in contrast to the manual column-base kit (Figure S11b and c). Using simulated human infections (un-infected human blood spiked with *T. brucei* parasites), we compared the performance of the *7SLRNA* SHERLOCK using DBS, whole blood and buffy coat. *7SLRNA* SHERLOCK detected trypanosome TNAs equivalent to 1 parasite/μL in the three types of samples (Fig. 3a), which is in line with the analytical sensitivity reported previously with the M18S-qPCR in DBS.^41^ To further quantify the *7SLRNA* SHERLOCK LoD, we analyzed 3 independent dilution series of 3 biological replicates of a simulated infection in whole blood and buffy coat samples. The estimated LoD was the lowest concentration where 3 out of 3 samples tested positive (Fig. 3b). Here, the resulting sensitivity was determined to be 10 parasites/μL in whole blood and 1 parasite/μL in buffy coat. This is consistent with the increased sensitivity seen with the mini-anion exchange centrifugation technique (mAECT) when buffy coat is analyzed instead of whole blood.^37^ This was resolved further for *7SLRNA* SHERLOCK using buffy coat and analysing 20 replicates of simulated infected samples at 0.66×, 1× or 1.5× a 1 parasite/μL parasitemia. We confirmed the *7SLRNA* SHERLOCK buffy coat LoD was 1 parasite/μL in 95% of the samples detected (Fig. 3c). Further improvement of the extraction methods will be required to increase the analytical sensitivity, since the LoD of SHERLOCK tests with RNA from cultured parasites was more sensitive (Fig. 1d).

### Assessment of the SHERLOCK4HAT diagnostic kit on cryobanked clinical samples

**Fig. 4:**
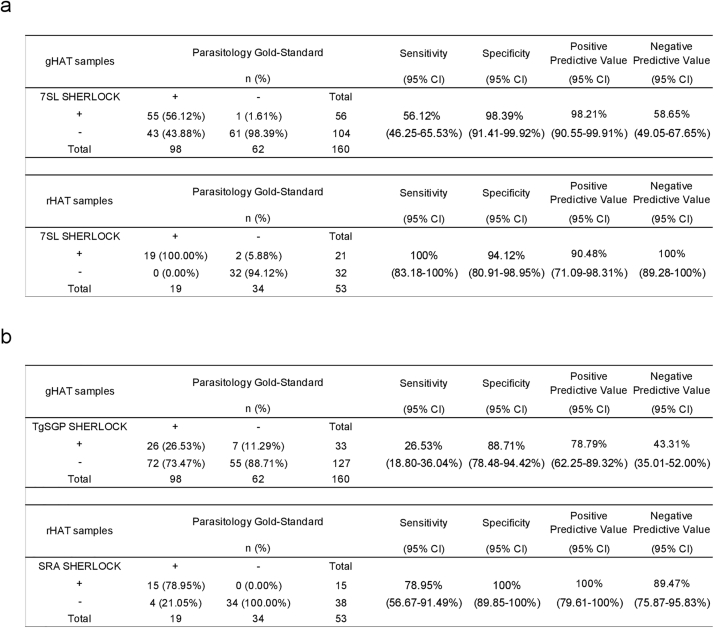
**Validation of SHERLOCK4HAT diagnostic using biobanked clinical samples.** Diagnostic performances of (a), subgenus-specific 7SL SHERLOCK and (b), sub-species specific TgSGP and SRA SHERLOCK assays on gHAT and rHAT buffy coat samples from the HAT WHO specimen Biobank. Concordance rates are shown as proportions of gold-standard diagnosis results. Sensitivity, specificity, positive and negative predictive values are assessed with a 95% confidence interval as compared to the initial parasitological gold-standard diagnosis performed prior to sample freezing. n, number of samples; CI, confidence interval.

**Fig. S12:**
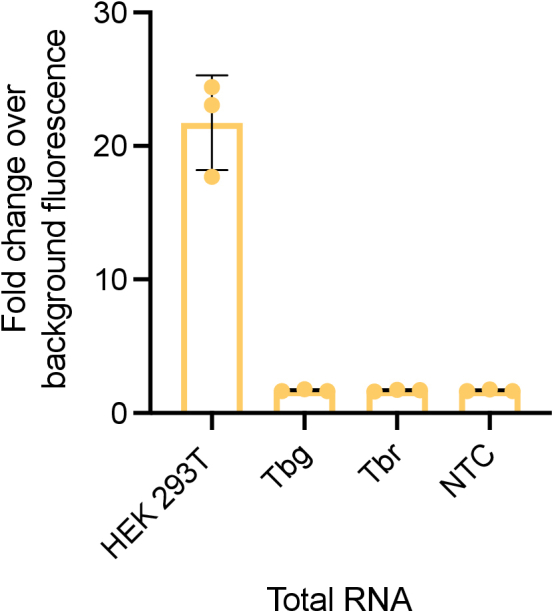
Human *RNase* P SHERLOCK assay. Total RNA from human cells (1 ng/μL) or cultured parasites (5 ng/μL) was assessed with *RNase P* SHERLOCK. *RNase P* test performed well and showed no cross-reactivity with parasite RNA. Bars represent the mean of three technical replicates ±SD.

**Fig. S13:**
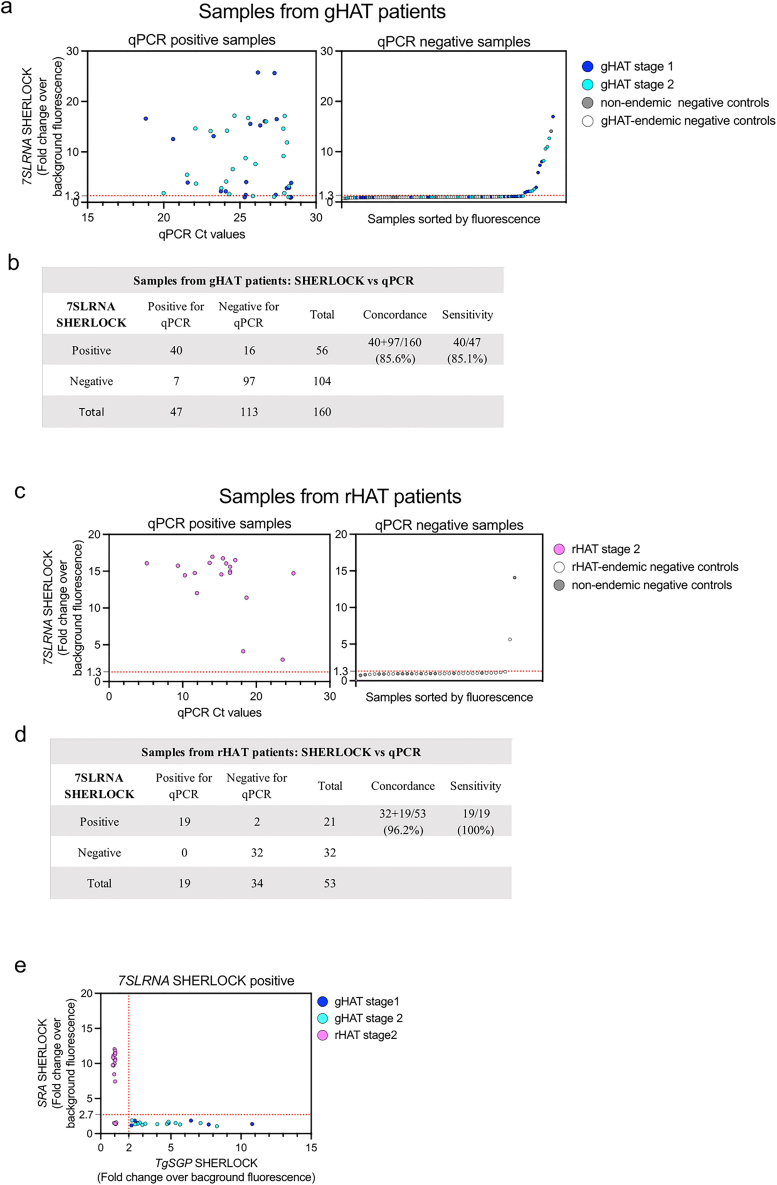
Validation of SHERLOCK4HAT diagnostic using bio-banked clinical samples. (a), *7SLRNA*SHERLOCK analysis of 160 gHAT qPCR positive (left) and negative (right) buffy coat samples obtained from human subjects in gHAT endemic and non-endemic regions. Each dot indicates one sample analysed by both *7SLRNA* SHERLOCK and *Tb177bp-repeats*-qPCR. The dashed red line is the threshold above which samples were considered positive for SHERLOCK. The dot colours indicate the classification according to the original diagnosis in the field. (b), Concordance tables, sensitivity and specificity of *7SLRNA* SHERLOCK for detection of trypanosome NA in buffy coat samples from individuals in gHAT endemic and non-endemic regions. qPCR indicates samples that were analysed by *Tb177bp-repeats-*qPCR. nC, negative controls from non-endemic regions, gC, gHAT negative endemic controls. (c), *7SLRNA* SHERLOCK analysis of 53 rHAT qPCR positive (left) and negative (right) buffy coat samples obtained from human subjects in rHAT endemic and non-endemic regions. Each dot indicates one sample analysed by both *7SLRNA* SHERLOCK and *Tb177bp-repeats*-qPCR. The dashed red line is the threshold above which samples were considered positive for SHERLOCK. The dot colours indicate the classification according to the original diagnostic in the field. (d), Concordance tables, sensitivity and specificity of *7SLRNA* SHERLOCK for detection of trypanosome NA in buffy coat samples from individuals in rHAT endemic and non-endemic regions. qPCR indicates samples that were analysed by *Tb177bp-repeats-*qPCR. nC, non-endemic negative controls, rC, rHAT endemic negative controls. (e), *TgSGP* and *SRA* SHERLOCK discriminate between *T. b. gambiense* and *T. b. rhodesiense* NA in patients. All gHAT and rHAT patient samples positive for *7SLRNA* were plotted according to their *TgSGP* and *SRA* SHERLOCK results. The thresholds for each target (red lines) were determined using ROC curve analyses of positive and negative sample data.

To validate SHERLOCK4HAT as a diagnostic tool kit, we used samples obtained from the WHO HAT specimen cryobank.^23^ Ninety-eight buffy coat samples from patients with confirmed gHAT; 48 buffy coat samples from gHAT endemic regions, but negative for gHAT, to act as controls; 19 buffy coat samples from patients with confirmed rHAT, and 20 buffy coat samples from rHAT endemic regions, but negative for rHAT, as further negative controls. Additionally, we analyzed 14 buffy coat samples from un-infected donors from non-endemic regions. For analysis, sample groups were defined by parasite species and parasitological results with 98 gHAT+, 62 gHAT−, 19 rHAT+ and 34 rHAT− (Fig. 4). As a positive technical control for TNA extraction to validate negative SHERLOCK results in clinical samples, i.e. to ensure that no SHERLOCK inhibitors were remaining in the sample, we designed an additional SHERLOCK assay that targeted the human *RNase P* gene and validated its performance using RNA from cultured human cells and parasites (Figure S12). For this analysis positivity is considered from a single test and all samples were positive for RNase P suggesting successful TNA extraction and no inhibition of the SHERLOCK reaction. The *7SLRNA* SHERLOCK detected trypanosomes in 55 out of 98 parasitologicaly confirmed gHAT samples with a sensitivity of 56.1%, 95% CI [46.25–65.53] (Fig. 4a). None of the gHAT endemic negative control samples tested positive, and one out of 14 non-endemic negative control samples was positive using the *7SLRNA* SHERLOCK with an overall specificity of 98.4%, 95% CI [91.41–99.92]. All 19 parasitologicaly confirmed rHAT patient samples tested positive for *7SLRNA* SHERLOCK with 100%, 95% CI [83.18–100] sensitivity and 94.1%, 95% CI [80.91–98.95] specificity (Fig. 4a and Data File S2). The *TgSGP* SHERLOCK detected 26.5% of the parasitologicaly confirmed samples with 88.7%, 95% CI [78.48–94.42] specificity and 26.5% 95% CI [18.8–36.04] sensitivity, reflecting the usually low parasitemia in gHAT patients (Fig. 4b). The *SRA* SHERLOCK detected trypanosomes in 79%, 95% CI [56.67–91.49] of the positive samples with 100%, 95% CI [89.95–100] specificity, in line with the usually high parasitemia in rHAT cases (Fig. 4b). Results of the 19 confirmed rHAT samples with *TgSGP* SHERLOCK were negative (Figure S13e). Likewise, 20 selected confirmed gHAT samples -positive for 7SLRNA and TgSGP SHERLOCK- were all negative when run using the *SRA* SHERLOCK diagnosis protocol (Fig. 4, Appendix Ae). Thus, the *TgSGP* and *SRA* SHERLOCK in patient samples accurately discriminate between *T. b. gambiense* and *T. b. rhodesiense* infections.

In addition to the expected natural species-specific differences in parasitemia, the variations in sensitivity between the species-specific and the pan-*Trypanozoon* SHERLOCK may also be caused by a selective degradation of the target RNAs. All samples were maintained at −80 °C from collection until delivery by the WHO HAT specimen biobank, and all the samples tested here were more than 10 years old and stored without any preservative.^23^ Therefore, the likelihood of TNA deterioration was high, especially for RNA. As an additional control, we ran a *Tb177 bp repeat* qPCR (Figure S13).^42^ Trypanosome DNA was detected by qPCR in 47 out of 98 confirmed gHAT samples (Figure S13a and Data File S2). The concordance between the two assays was 85.6%, with 40 out of 47 qPCR positive samples and 97 out of 113 qPCR negative samples, positive and negative for SHERLOCK respectively (Figure S13a and b and Data File S2). In addition, 15 of the 16 qPCR negative samples that tested positive for SHERLOCK were part of the originally confirmed gHAT cohort, revealing our SHERLOCK diagnostic to be more sensitive than the qPCR test (Figure S13a and b and Data File S2). The sensitivity of the *7SLRNA* SHERLOCK was 85.1% for gHAT, based on the qPCR positive samples (Figure S13b and Data File S2). The *TgSGP* SHERLOCK detected 42.5% of the qPCR positive samples with 88.7% specificity (Data File S2 and Table S7). Higher parasitemia in *T. b. rhodesiense* infections^1^ were also observed here with lower qPCR Ct values in rHAT samples compared to those in gHAT samples (Figure S13c and d and Data File S2). The *SRA* SHERLOCK detected 79% of the qPCR positive samples with 100% specificity (Data File S2 and Table S7). No correlation between the stage of the disease and the qPCR Ct values or the SHERLOCK fluorescence readouts in gHAT patients was observed (Data File S2).

## Discussion

Here we described the development of a new molecular detection toolkit for both HAT diagnosis and epidemiological surveillance. Our SHERLOCK4HAT diagnostic can distinguish between the three *T. brucei* subspecies using a pan-*Trypanozoon*, *gambiense*-specific, or *rhodesiense*-specific targets. Although our subspecies-specific targets use *TgSGP* and *SRA*, which are related to *VSG* genes, we do not see cross reactivity. In fact, in spite of the degree of DNA sequence homology shared between *SRA* and *VSG* variants, we only saw one false positives in a *T. b. gambiense* sample, confirming that the selected target meets the specificity requirements for rHAT diagnosis.

As an RNA based diagnostic, SHERLOCK4HAT is a highly sensitive and species-specific detection method for on-going infections with a simple set up. Using *in vitro* transcribed RNA, the LoD was calculated to be 200 aM (100 molecules/μL) for *7SLRNA* and *TgSGP*, and 20 aM (10 molecules/μL) for *SRA* (Figure S7). This analytical sensitivity is similar to that reported previously for other molecular diagnostics that are subgenus-specific^41^, ^42^, ^43^, ^44^, ^45^, ^46^, ^47^ and 10 to 100-fold more sensitive to those reported for subspecies-specific tests.^33^^,^^48^^,^^49^ Using blood spiked with parasites, we show that the analytical sensitivity of SHERLOCK4HAT for the *Trypanozoon* target is 1 parasite/μL (1000 parasites/mL), which is comparable to several molecular techniques for detection of *Trypanozoon* taxa (mAECT on blood at 50 trypanosomes/mL, TBR-PCR/qPCR and 18S-PCR at 50–100 parasites/mL).^41^, ^42^, ^43^^,^^50^ However, implementation of these techniques is limited by the need for sophisticated equipment. An additional advantage of SHERLOCK4HAT is the single temperature isothermal RPA amplification coupled to a Cas13 detection, making our method more adapted to the low-income countries where the disease is endemic. Other isothermal approaches have been developed with similar sensitivities to SHERLOCK (LAMP-100 parasites/mL, NASBA-10 parasites/mL),^49^^,^^51^ but significant infrastructure costs have limited their implementation in control programs. Our subspecies-specific SHERLOCK4HAT diagnostics using a *TgSGP* or *SRA* target are 10 to 100-fold more sensitive than that the current subspecies-specific diagnostics using PCR/qPCR^33^^,^^48^^,^^52^ and show no overlap between the signal in positive and negative samples, resolving any ambiguity seen in PCR and qPCR. In fact, our results indicated that SHERLOCK4HAT can unequivocally discriminate between *TgSGP* and *SRA,* and therefore diagnose of *T. b. gambiense* and *T. b. rhodesiense* infections.

The current gHAT field-applicable diagnostic algorithms are based on antibody detection in patient blood (CATT or RDTs) with confirmation of seropositive cases by parasitological observation.^1^^,^^2^^,^^4^^,^^5^ These methods present some limitations: (i) false-negative results if the *VSG* variants detected by the test are poorly or not expressed,^53^^[preprint]^ (ii) reduced specificity,^1^^,^^2^^,^^4^^,^^5^ and (iii) relatively high cost (RDTs), significant workload (CATT on serial plasma dilution, time at microscope) and need for specialized staff and equipment (especially for parasitological confirmation). For rHAT, no field-applicable diagnostic methods exist. SHERLOCK4HAT overcomes these limitations. We have shown that SHERLOCK4HAT detection is not limited by geography or time after sampling using biobanked samples, it is easy to set up, does not require sophisticated equipment and it is adapted for high-throughput applications (fluorescence readout) or individual testing (LFA), making it versatile for both surveillance at reference centres and eventually for PoC testing, after further technical optimization. The SHERLOCK4HAT diagnostic can be run in 1 h 30 min for a one-step reaction (at 4 € if coupled to a commercial LFA, exclusive of sample processing), or 2 h 30 min for a two-step reaction (at 2.5 €, exclusive of sample processing) (Table S6), and these costs would be notably reduced with an in-house manufactured strip for LFA.

One limitation for SHERLOCK4HAT, as for any molecular diagnostic method, is the NA extraction step. Several extraction methods coupled to a CRISPR-based diagnostic reaction have been published,^10^^,^^54^^,^^55^ but remained to be tested in the context of HAT diagnostics. For high-throughput surveillance using SHERLOCK4HAT, automated NA extraction systems can be implemented with higher reproducibility, reduced hand-on time and no cross-contamination. Manual extraction methods, although more time consuming, showed an increased analytical sensitivity which is consistent with previous studies.^56^ As an RNA-based diagnostic, SHERLOCK is limited by the increased sensitivity of RNA to nuclease degradation, which can affect the sensitivity of the test if the clinical specimens are not stored properly. Nucleic acid stabilization buffers or Flinders Technology Associates (FTA) cards to transport and store the samples can be used to attenuate these limitations. It should be noted that RNA is a better indicator of active infections than DNA,^47^ making SHERLOCK4HAT a valuable tool for assessing treatment outcome.

The barely detectable parasitaemia characterising gHAT may explain that 44% of confirmed gHAT patient samples were negative with SHERLOCK, and that 53% were also negative using standard qPCR analysis. Nevertheless, the discrepancy between these results compared to the original in-field diagnostic could also be due, at least in part, to the selective deterioration of the nucleic acids in these samples, that were stored at −80 °C for more than 10 years without preservative.^23^ Low parasitemia is typical in gHAT infections, thus any NA degradation could have a dramatic effect on detection using molecular techniques. *T. b. rhodesiense* infections have higher parasitemia, hence deterioration of NA in the samples might have a lower impact in the diagnostic sensitivity, which is evident given the robust sensitivity using rHAT SHERLOCK. The lower sensitivity observed with the *TgSGP* target (26.5%) could be attributed to a selective degradation of the target RNA and/or to a differential expression of the *TgSGP* gene in these samples, since the analytical sensitivity of SHERLOCK for *7SLRNA* and *TgSGP* was similar. From the 62 negative control samples, 7 tested positive for *TgSGP* compared to 1 or 0 for *7SLRNA* SHERLOCK or qPCR, respectively. This reduced specificity needs to be interrogated further.

As we move towards the elimination phase of gHAT, if not to the post-elimination phase in several countries, SHERLOCK4HAT could certainly be a viable surveillance tool, as well as a possible alternative for PoC diagnostic test once it would have been technically optimized on purpose. Optimization of the one-pot reaction to meet the sensitivity requirements for HAT diagnosis, lyophilization of the reaction components and field-friendly NA extraction methods will be required before large-scale deployment. Sensitivity could also be improved using a combination of *7SLRNA* and *TgSGP* or *SRA* targets in a multiplex SHERLOCK4HAT diagnostic kit that would allow the detection of the two human infective subspecies of *T. brucei* at the same time in a single reaction, thereby reducing the full diagnostic cost and making the technology more adapted for horizontal epidemiological studies, including in animal reservoirs. In total, SHERLOCK4HAT provides a readily adaptable diagnostic method for HAT, mass screening and epidemiological surveillance.

## Contributors

NS, BR and LG conceived and designed the experiments. NS, ADH performed the experiments. NS, BR and LG verified and analyzed the data. MNU, BLP, BR and LG contributed reagents, materials and analysis tools. NS, BR and LG wrote the paper. All authors read and approved the final version of the manuscript.

## Data and materials availability

All data are available in the main text or the supplementary materials.

## Declaration of interests

The authors declare that they have no competing interests.

## Figures and Tables

**Fig. S8:**
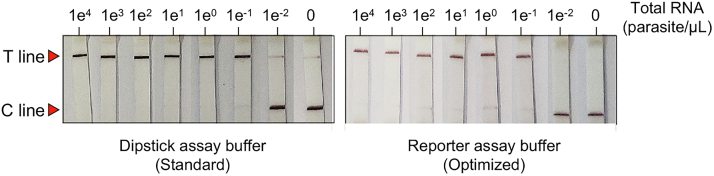
Lateral Flow Assay (LFA) optimization. Performance of two LFA buffers. A reporter assay buffer or a dipstick assay buffer were used to dilute the *7SLRNA* SHERLOCK reaction prior the lateral flow assay. The signal was read after 5 min of incubation. Left panel is the experiment shown in Fig. 1e for comparison.

## Appendix A Supplementary data

Supplementary Western blot

Caption for Supplementary Material

Data File S1 BLAST hits of *7SLRNA* and *SODB1* sequences. Hits obtained running the *7SLRNA* (Tb927.82861) and *SODB1* (Tb927.11.15910) sequences on the *Trypanozoon* nucleotide data base using BLASTn from the National Center for Biotechnology Information (NBCI).

Data File S2 WHO HAT specimen biobank clinical samples. Individual information, SHERLOCK4HAT and qPCR results.

Table S1 RPA primers and crRNA templates. Selected RPA primer pairs and crRNAs for each target gene are highlighted.

Table S2 Single nucleotide polymorphisms identified in the genes of this work.

Table S3 Other oligonucleotides.

Table S4 RNA samples from the trypanosome biobank at ITM and SHERLOCK analysis. BSF, bloodstream form; PCF, procyclic form; DRC, Democratic Republic of Congo; ITM, Institute of Tropical Medicine, Antwerp, Belgium; NA, (information) not available; ∗ RNAs extracted from different species, strains and/or stabilates, either freshly isolated, or maintained in animal models and/or cultured in synthetic media with different durations and numbers of passages, and therefore theoretically presenting different transcriptomes.

Table S5 SHERLOCK performance of the screened crRNAs. Background subtracted fluorescence (a. u.) ≤1500, no signal; Background subtracted fluorescence (a. u.) >1500 and ≤5000, Low; Background subtracted fluorescence (a. u.) >5000 and ≤15000, Medium; Background subtracted fluorescence (a. u.) >15000, High; NA, not assessed; Tbb, *T. b. bucei*; Tbg, *T. b. gambiense*; Tbr, *T. b. rhodesiense*; sspp, subspecies.

Table S6 Cost analysis of a SHERLOCK4HAT unit.

Table S7 Concordance tables, sensitivity and specificity of SHERLOCK4HAT diagnostic kit for detection of trypanosome nucleic acids in buffy coat samples.
