# Supplementary figures and images for: SHERLOCK4HAT: A CRISPR-based tool kit for diagnosis of Human African Trypanosomiasis
Source: eBioMedicine. 2022 Oct 27;85:104308. doi: 10.1016/j.ebiom.2022.104308 (PMC9626900; doi:10.1016/j.ebiom.2022.104308)

## Slide 1
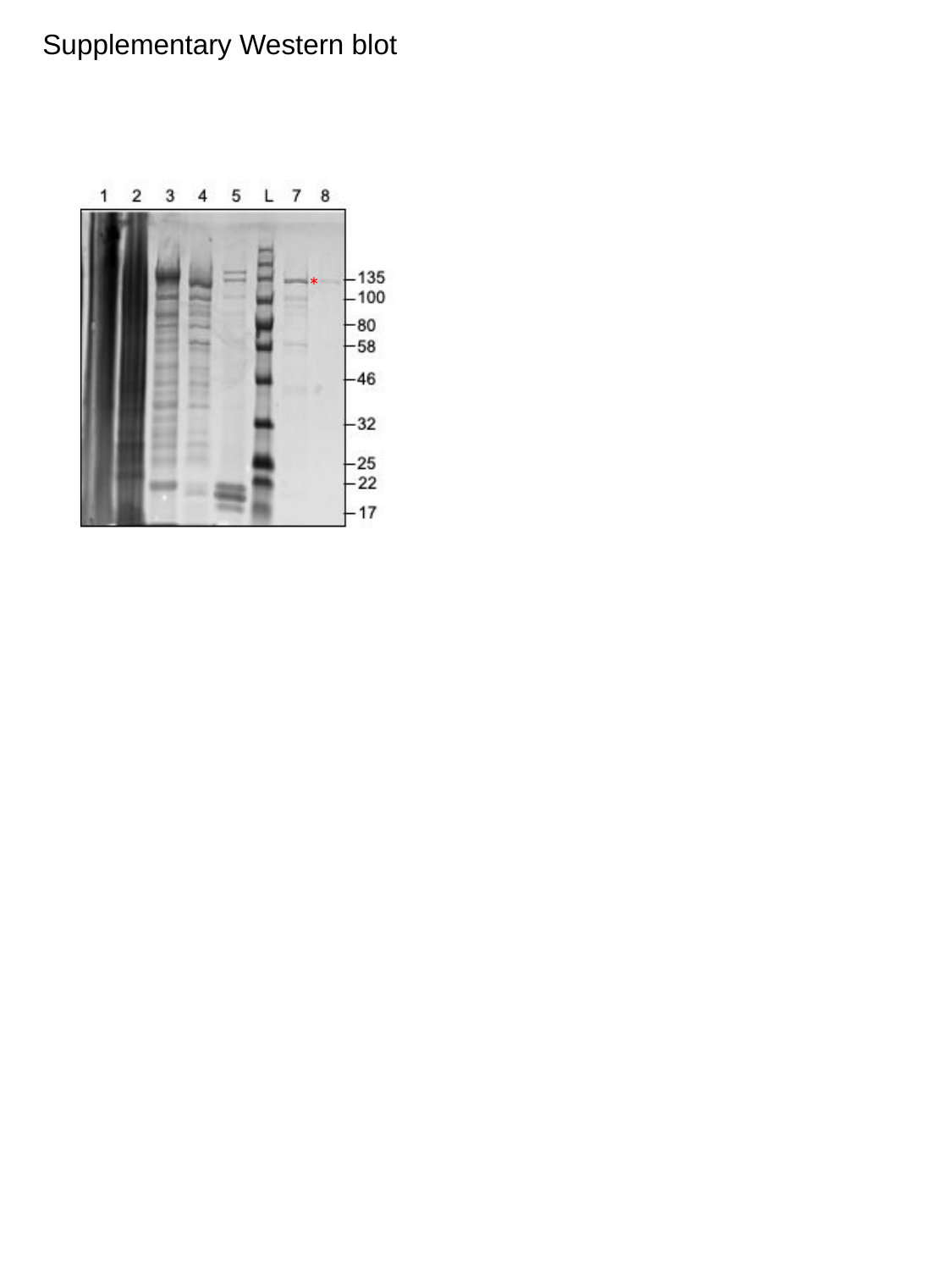

Supplementary Western blot
*

Supplement: Supplementary Western blot [file mmc1.pptx]
